# Impacts of Saharan Mineral Dust on Air‐Sea Interaction over North Atlantic Ocean Using a Fully Coupled Regional Model

**DOI:** 10.1029/2020JD033586

**Published:** 2021-02-17

**Authors:** Shu‐Hua Chen, Chu‐Chun Huang, Yi‐Chun Kuo, Yu‐Heng Tseng, Yu Gu, Kenneth Earl, Chih‐Ying Chen, Yonghan Choi, Kuo‐Nan Liou

**Affiliations:** ^1^ Department of Land, Air, and Water Resources University of California Davis CA USA; ^2^ Institute of Oceanography National Taiwan University Taipei Taiwan; ^3^ Joint Institute for Regional Earth System Science and Engineering and Department of Atmospheric and Oceanic Sciences University of California Los Angeles CA USA; ^4^ NASA Goddard Space Flight Center Greenbelt MD USA; ^5^ Research Center for Environmental Changes Academia Sinica Taipei Taiwan; ^6^ Korea Polar Research Institute Incheon South Korea

**Keywords:** African dust, air‐sea interaction, COAWST, dust‐cloud‐radiation interaction, mixed layer depth, sea surface temperature, surface heat fluxes, wind stress curl

## Abstract

This study examines the modifications of air‐sea coupling processes by dust‐radiation‐cloud interactions over the North Atlantic Ocean using a high‐resolution coupled atmosphere‐wave‐ocean‐dust (AWOD) regional model. The dust‐induced mechanisms that are responsible for changes of sea surface temperature (SST) and latent and sensible heat fluxes (LHF/SHF) are also examined. Two 3‐month numerical experiments are conducted, and they differ only in the activation and deactivation of dust‐radiation‐cloud interactions. Model results show that the dust significantly reduces surface downward radiation fluxes (SDRF) over the ocean with the maximum change of 20–30 W m^−2^. Over the dust plume region, the dust effect creates a low‐pressure anomaly and a cyclonic circulation anomaly, which drives a positive wind stress curl anomaly, thereby reducing sea surface height and mixed layer depth. However, the SST change by dust, ranging from −0.5 to 0.5 K, has a great spatial variation which differs from the dust plume shape. Dust cools SST around the West African coast, except under the maximum dust plume ridge, and extends westward asymmetrically along the northern and southern edges of the dust plume. Dust unexpectedly warms SST over a large area of the western tropical North Atlantic and north of the dust plume. These SST changes are controlled by different mechanisms. Unlike the SST change pattern, the LHF and SHF changes are mostly reduced underneath the dust plume region, though they are different in detail due to different dominant factors, and increased south of the dust plume over the tropic.

## Key Points

- Dust‐radiation‐cloud interactions mainly cool SST offshore the North Africa and warm SST over the southwestern dust plume region
- Dust reduces upward LHF and SHF underneath the dust plume region and increases the values south of the dust plume
- Changes of SST/LHF/SHF by dust are not only attributed to the dust‐induced SDRF reduction but also changes of WSC and upper‐ocean dynamic

## 1 Introduction

The atmosphere and oceans act as a coupled system. They interact in various ways, including via ocean‐surface wind stresses, downward radiation fluxes, and exchanges of surface heat/energy. These interactions are critical for driving ocean currents and large‐scale circulation patterns, modulating upper‐ocean dynamics and thermodynamics, controlling atmospheric cloud development, and shaping climate and weather phenomena (e.g., Cronin et al., 2019; Yu, 2019; Zhang, 2005).

The oceans, which represent the largest reservoir of energy in the climate system, exchange energy with the atmosphere in the form of sensible and latent heat fluxes (SHF/LHF). Upward heat fluxes from the ocean‐surface destabilize the lower troposphere and promote cloud development (Yu, 2009). Both SHF and LHF are modulated by several properties at or near the ocean‐surface, including sea surface temperature (SST).

SSTs can strongly affect atmospheric features across a range of spatiotemporal scales, including tropical cyclones (TC; Magnusson et al., 2014; Wang et al., 2010), intraseasonal weather patterns (Sobel et al., 2010; Wang et al., 2018; Zhang et al., 2018), and climate (Dittus et al., 2018; Li & Sriver, 2018). Cione and Uhlhorn (2003) demonstrate that a 1 °C decline in SSTs can reduce the surface enthalpy flux (LHF plus SHF) by ∼40% within the inner core of a TC. They also demonstrate a statistical correlation between SST variability and TC‐intensity fluctuations. The Madden‐Julian oscillation (MJO), which is the primary driver of intraseasonal variability in the tropics, is characterized by the interplay between tropical convection, surface heat fluxes, and SSTs (Madden & Julian, 1971; Woolnough et al., 2000; Zhang, 2005). Peak convective activity in the MJO commonly occurs about 10 days after the development of a warm SST anomaly (Maloney & Kiehl, 2002). On an even greater temporospatial scale, the feedback between zonal SST gradients and surface winds across the equatorial Pacific Ocean is a defining feature of the El Niño/Southern Oscillation, which is responsible for most of the interseasonal‐interannual variability in the tropical Pacific region (Timmermann et al., 2018).

Since air‐sea interactions influence weather systems and climate, it is important to accurately represent them in numerical models in order to improve forecasts and simulations. Atmosphere‐ocean coupled models, which directly simulate air‐sea interactions, have been shown to better predict extreme weather (Di Sante et al., 2019; Hirons et al., 2018; Li & Sriver, 2018) and intraseasonal and interseasonal variations in Indian summer monsoon rainfall (Di Sante et al., 2019; Li & Sriver, 2018; Misra et al., 2017; Wang et al., 2018) than do numerical models that rely on predefined SSTs to represent the ocean's influence on the atmosphere. This is primarily because fully coupled ocean‐atmosphere models: (1) ensure that the atmosphere and oceans are dynamically and physically consistent, and (2) update the required status/fluxes more frequently to correctly simulate air‐sea interactions.

Air‐sea interactions can also be affected by atmospheric dynamics and thermodynamics, including those impacted by aerosol‐radiation‐cloud processes. Mineral dust aerosols absorb and scatter incoming solar radiation, generally increasing the temperature within a dust layer, cooling the near‐surface layer below, and reducing the surface downward shortwave (SW) radiation flux. Additionally, dust also absorbs, scatters, and emits longwave (LW) radiation, cooling the upper portions of a dust layer, warming the atmosphere below, and increasing the surface downward LW radiation flux. These direct radiative effects modify atmospheric temperature stratification and thus atmospheric instability, atmospheric circulations, cloud development, and precipitation (the semidirect effect) (Bangalath & Stenchikov, 2015; Kok et al., 2018; Miller & Tegen, 1998; Yoshioka et al., 2007). Dust can also serve as cloud condensation nuclei (CCN) (Koehler et al., 2009; Kumar et al., 2009) and ice nuclei (IN) (Atkinson et al., 2013; DeMott et al., 2003; Twohy et al., 2009), thereby affecting cloud properties such as droplet number concentration, coverage, and cloud top temperature that in turn alter cloud‐radiation interactions (Gibbons et al., 2018; Karydis et al., 2017; Li et al., 2017; Twohy et al., 2017). Together, dust‐radiation‐cloud effects can impact the ocean by altering near‐surface characteristics (e.g., wind and surface radiation flux) (Foltz & McPhaden, 2008; Guo & Yin, 2015; Lau & Kim, 2007; Martínez Avellaneda et al., 2010). Since the atmosphere and oceans are intrinsically linked, dust‐driven changes to the ocean could in turn impact atmospheric circulation patterns, weather, and climate.

The Sahara Desert is the most active dust source in the world (Prospero et al., 2002). From late spring to early fall, dust plumes appear regularly over the desert and downstream over the Atlantic Ocean. Saharan dust affects both cloud processes and local radiation budgets, modifying weather systems and large‐scale circulation patterns over North Africa and the Atlantic Ocean, including TCs (e.g., Chen et al., 2015;Dunion & Velden, 2004; Pan et al., 2018), mesoscale convective systems (e.g., Huang et al., 2019; Martínez & Chaboureau, 2018; Shi et al., 2014), the West African monsoon (e.g., Konare et al., 2008; N'Datchoh et al., 2018; Zhao et al., 2011), the African Easterly Jet (AEJ), and African Easterly Waves (AEW) (e.g., Bercos‐Hickey et al., 2017; Grogan & Thorncroft, 2019; Nathan et al., 2017).

It has been shown that Saharan dust can affect SSTs, LHFs, and SHFs over the Atlantic Ocean (Evan et al., 2008; Jordan et al., 2018; Strong et al., 2015; Yue et al., 2011). Many studies have attempted to quantify the impact of dust aerosols on SSTs and upper‐ocean dynamics using atmosphere‐ocean coupled numerical models, most of which have employed simplified ocean models including mixed layer models (Lau et al., 2009; Miller et al., 2014) or slab (Mahowald et al., 2010; Yoshioka et al., 2007) that neglect ocean horizontal advection and the impact of the wind stress curl (WSC). A pair of recent studies has, however, used a full three‐dimensional ocean model in an atmosphere‐ocean coupled model to examine the impact of dust on the upper ocean, weather, and climate over North Africa and the tropical Atlantic Ocean (Jordan et al., 2018; Strong et al., 2015). Both studies are discussed below.

Strong et al. (2015) conducted 150‐year climate simulations to examine and compare the impact of dust on West African monsoon rainfall and upper‐ocean characteristics using two different sets of dust‐optical properties: one with greater absorption (ABS) and the other with greater scattering (SCT). They used the fully coupled Geophysical Fluid Dynamics Laboratory (GFDL) climate model with a horizontal grid resolution of 2° × 2.5° for the atmospheric component and a nominal resolution of 1°^ ^× 1° for the ocean component. Dust was found to increase West African monsoon rainfall and shift the intertropical convergence zone (ITCZ) northward in the ABS case and do the exact opposite in the SCT case. On the other hand, both simulations consistently reduce the surface downward radiation fluxes (SDRF) with a larger reduction in the ABS case (∼30 W m^−2^ in ABS versus 20 W m^−2^ in SCT). In the upper ocean, the two simulations once again produce opposite results, with ABS dust reducing and SCT dust increasing upper‐ocean heat content. They conclude that this is caused by changes in the WSC due to the dust‐driven latitudinal displacement of the ITCZ and the accompanying changes to the mixed layer depth within the region.

Several years later, Jordan et al. (2018) examined the impacts of dust on summertime rainfall over North Africa and East Atlantic in both fully coupled and uncoupled 1800‐year GFDL ocean‐atmosphere model simulations. In the fully coupled simulation, dust significantly reduces the net SDRF beneath the dust plume by up to 30 W m^−2^, reduces SST particularly beneath the southern part of the dust plume, reduces the LHF, decreases sea level pressure by ∼0.5 hPa, and induces a cyclonic circulation anomaly near the surface. Rainfall increases over North Africa but decreases over the eastern Atlantic Ocean due to dust. Their rainfall results are similar to those of Miller et al. (2014). In the uncoupled simulation, however, dust increases rainfall over both land and ocean. They posited that differences of rainfall changes by dust between the two simulations (i.e., coupled versus uncoupled) were due to the reduced SDRF over the ocean and enhanced static stability created by dust‐induced temperature changes in the coupled simulation. Note that the simulations of both Strong et al. (2015) and Jordan et al. (2018) used a prescribed dust distribution field and ignored dust‐cloud interactions (i.e., the dust indirect effect).

While both Strong et al. (2015) and Jordan et al. (2018) examined the effects of dust on the ocean‐atmosphere system using fully coupled, three‐dimensional ocean and atmosphere models, both relied on coarse‐resolution global models that did not include dust‐cloud effects, and both used prescribed—rather than predicted—dust fields. In this study, we seek to contribute to the current understanding of how dust‐physical processes influence air‐sea interactions and upper‐ocean currents using high‐resolution numerical experiments carried out with a fully coupled, three‐dimensional atmosphere‐ocean‐dust regional model in which the three‐dimensional dust field is entirely predicted. A follow‐up work, using the same numerical data, will examine the impact of dust‐altered air‐sea interactions on the West African monsoon and the ITCZ.

The paper is organized as follows. Section 2 introduces the new coupled atmosphere‐ocean‐dust model, numerical experiment design, and data used. Section 3 examines the impact of dust on surface radiation fluxes, low‐level meteorological variables, and upper‐ocean dynamics, while section 4 analyzes the impact of dust on SSTs, air‐sea interactions, and mechanisms. We conclude the paper with a summary and discussion in section 5.

## 2 Numerical Model and Experimental Design

### 2.1 Model description

The impacts of dust on air‐sea interactions are examined using a fully coupled Atmosphere‐Wave‐Ocean‐Dust (AWOD) model. Our AWOD model integrates two existing model components: The Coupled Ocean‐Atmosphere‐Wave‐Sediment Transport (COAWST) model (Warner et al., 2008, 2010), which was developed at the United States Geological Survey (USGS), and the online atmosphere‐dust model by Chen et al. (2010, 2015).

The COAWST model consists of the atmospheric Weather Research and Forecasting (WRF) model (Skamarock et al., 2008), the Regional Ocean Modeling System (ROMS, Shchepetkin & McWilliams, 2005), and the Simulating WAves Nearshore (SWAN) wave model with the Sediment Transport Model capability (Warner et al., 2010). All model components in COAWST are widely used for a broad range of regional applications. To emphasize the interaction between the ocean and atmosphere, only the oceanic and atmospheric components are used in this study. WRF provides surface energy flux and wind stress information to ROMS, which in turn provides SST information to WRF.

The online atmosphere‐dust model, the WRF‐Dust model, adds a dust‐continuity equation and dust‐physical processes, including dust‐radiation‐cloud interactions, to the WRF model (Chen et al., 2010, 2015; Huang et al., 2019). The use of WRF in both the COAWST and WRF‐Dust models facilitated the process of integrating the two. In the AWOD model, dust affects the ocean by changing the SDRF and near‐surface meteorological variables (i.e., temperature, moisture, and wind). Dust‐induced SST changes can then affect the atmosphere via the lower boundary of the WRF model. Note that dust is entirely omitted from the ocean model component, so the physics of dust within the ocean, as well as atmosphere‐ocean exchanges of dust, are not considered in this study. Below we briefly describe the dust module found in both the AWOD and WRF‐Dust models (Chen et al., 2010, 2015; Huang et al., 2019).

The dust particle size distribution is represented by five size bins centered at radii of 0.25, 0.5, 1, 2, and 4 μm. In the AWOD model, the amount of dust in each size bin is governed by a continuity equation, which is written as:

(1) $$\frac{\partial \mu \gamma }{\partial t}=\nabla \cdot \vec{V}\mu \gamma +\gamma_{pbl}+\gamma_{con}+\gamma_{mic}+\gamma_{pbl}+S_{\gamma }+E_{\gamma },$$

where *γ* is the dust mixing ratio (kg kg^−1^); $\vec{V}$ is the three‐dimensional wind vector; and *μ *= (*p*_*s *_−* p*_*t*_) represents the column mass, computed as the hydrostatic pressure difference between the surface (*p*_*s*_) and the top (*p*_*t*_) of the WRF model. The first term on the right‐hand side of (1) is the flux divergence of dust; $\gamma_{pbl}$ is the subgrid boundary layer mixing; $\gamma_{con}$ represents the subgrid cumulus mixing; $\gamma_{mic}$ is a sink/source term for microphysical processes (e.g., dust activated as cloud condensation nuclei, wet scavenging, etc.); $S_{\gamma }$ denotes dry deposition; and $E_{\gamma }$ is surface emission. In this study, surface dust emission occurs in any grid cell where the land surface classification is “barren,” the soil volumetric moisture is less than 0.2, and the 10‐m wind speed exceeds a threshold velocity of 6.5 m s^−1^. It is computed by multiplying the formulation of Tegen and Fung (1994) by the erodibility factor of the WRF chemistry model (Ginoux et al., 2001). The full dust emission flux is then partitioned into each size bin according to Kok (2011).

The impact of dust‐radiation interactions is introduced in the diabatic heating term of the atmospheric thermodynamic equation and quantified in the SW and LW radiation parameterizations. In this study, the Fu‐Liou‐Gu (FLG) radiation scheme (Gu et al., 2010, 2011) is chosen. The FLG radiation scheme is a modified and improved version based on the Fu‐Liou radiative transfer model (Fu & Liou, 1992, 1993). A combination of the delta four‐stream approximation for solar flux calculations (Liou et al., 1988) and a delta‐two‐four‐stream approximation for thermal infrared flux calculations (Fu et al., 1997), which are divided into 6 and 12 bands, respectively, was implemented in the scheme. The radiative effects of 18 aerosol types, 5 of which are mineral dust, are computed using pretabulated, spherical‐particle Lorenz‐Mie single‐scattering properties interpolated from the Optical Properties of Aerosols and Clouds (OPAC) database (D'Almeida et al., 1991; Hess et al., 1998; Tegen & Lacis, 1996) to the 18 spectral bands of the FLG scheme (Gu et al., 2006). The FLG scheme has been extensively used to assess aerosol radiative effects in regional and global climate models (Gu et al., 2006, 2010, 2012; Zhao et al., 2016, 2017). Note that we only consider the radiative effects of mineral dust in this study.

S.‐H Chen et al. (2015) implemented a two‐moment microphysics scheme (Cheng et al., 2010) into the WRF‐Dust model. The scheme has been modified to include dust‐cloud interactions and has been tested in Huang et al. (2019). Dust can act both as IN via immersion freezing and deposition (Chen et al., 2008; Hoose et al., 2010), and CCN via adsorption (Kumar et al., 2011a, 2011b; Navea et al., 2010). The hygroscopicity parameter, κ, is set to 0.05 based on Koehler et al. (2009). The critical radius for dust‐CCN activation and the equilibrium size of activated cloud drops are based on J.‐P Chen et al. (2013).

### 2.2 Experimental Designs

In general, the ocean has a longer memory than the atmosphere in response to an external forcing. Thus, a seasonal study, rather than a case study, is conducted here to evaluate the impact of mineral dust on air‐sea interactions and upper‐ocean characteristics over the East and Central Atlantic. Two AWOD model experiments are conducted, differing only by the activation (DON) or deactivation (DOFF) of dust‐radiation‐cloud interactions. Both experiments are integrated from June 1, 2015 to September 30, 2015, with the month of June treated as a spin‐up period that is excluded from the analysis. The remaining three months, which span the climatological peak in Saharan dust activity, are chosen for evaluation. These are also the wettest months of the West African Monsoon, which is strongly modulated by air‐sea interactions, though this part of the work will be presented in a separate study. We note that the observed aerosol optical depth during these three months in the year of 2015, mainly contributed by dust, is close to the climatological average (figure not shown), so that the chosen time frame is generally representative.

In the AWOD model, only one domain is configured for both the atmosphere (i.e., WRF) and ocean (i.e., ROMS) components with approximately the same horizontal grid spacing (15 km) and the same domain size, covering from about 15°S to 43°N and 32°E to 68°W. The vertical grid spacing is stretched with higher resolutions near the surface for both atmosphere and ocean. The use of a 15 km horizontal grid spacing or finer is essential in order to resolve atmospheric weather systems that produce high surface winds, which are responsible for uplifting Saharan dust over the desert. Moreover, simulating at this resolution (∼15 km) can allow for oceanic fronts and mesoscale features to develop in the Atlantic, such as the tropical instability waves, mesoscale eddies and coastal upwelling. The eddy kinetic energy in the ocean can be even larger than the mean kinetic energy in the tropics (e.g., Seo & Xie, 2011; Tubul et al., 2015; Wu & Bowman, 2007) and significantly affect the air‐sea fluxes. Indeed, the mesoscale feature plays a dominant role on the ocean‐atmosphere interaction (Small et al., 2008, 2014), which cannot be ignored in an accurate model simulation.

The initial and boundary conditions of both DON and DOFF simulations are from six‐hourly Climate Forecast System (CFS) version 2 analysis (CFSv2; ∼1° × 1° spatial resolution) for the atmosphere and 1/12° Hybrid Coordinate Ocean Model (HYCOM) analysis (updated every 10 days) for oceans. The physics parameterizations used in the atmospheric component include the Medium Range Forecast (MRF) boundary layer scheme (Hong & Pan, 1996), the Kain‐Fritsch (KF) cumulus scheme (Kain, 2004), the FLG radiation scheme (Gu et al., 2010, 2011), and a two‐moment microphysics scheme (Cheng et al., 2010; Huang et al., 2019); all of these schemes account for dust‐physical processes. For the ocean, we use the Generic Length Scale (GLS) vertical mixing scheme (Umlauf & Burchard, 2003).

## 3 Simulation Results and Discussion

As mentioned in Section 1, the SST plays an important role in air‐sea interactions, which are controlled by different processes, including but not limited to surface energy fluxes, advection, and vertical mixing in the upper‐ocean layer. Before investigating the influence of dust‐radiation‐cloud interactions on SST, air‐sea interactions, and upper‐ocean currents, we evaluate the performance of the AWOD model. We then assess the impacts of dust on near‐surface meteorological variables and upper‐ocean processes.

In the following discussion, three‐hourly model output data from July 1 to September 30, 2015 are used for comparison between DON and DOFF. We note that data at different time frequencies might be used to compare with observational or reanalysis fields depending on their availability.

### 3.1 Evaluation of the Model Performance

**Figure 1:**
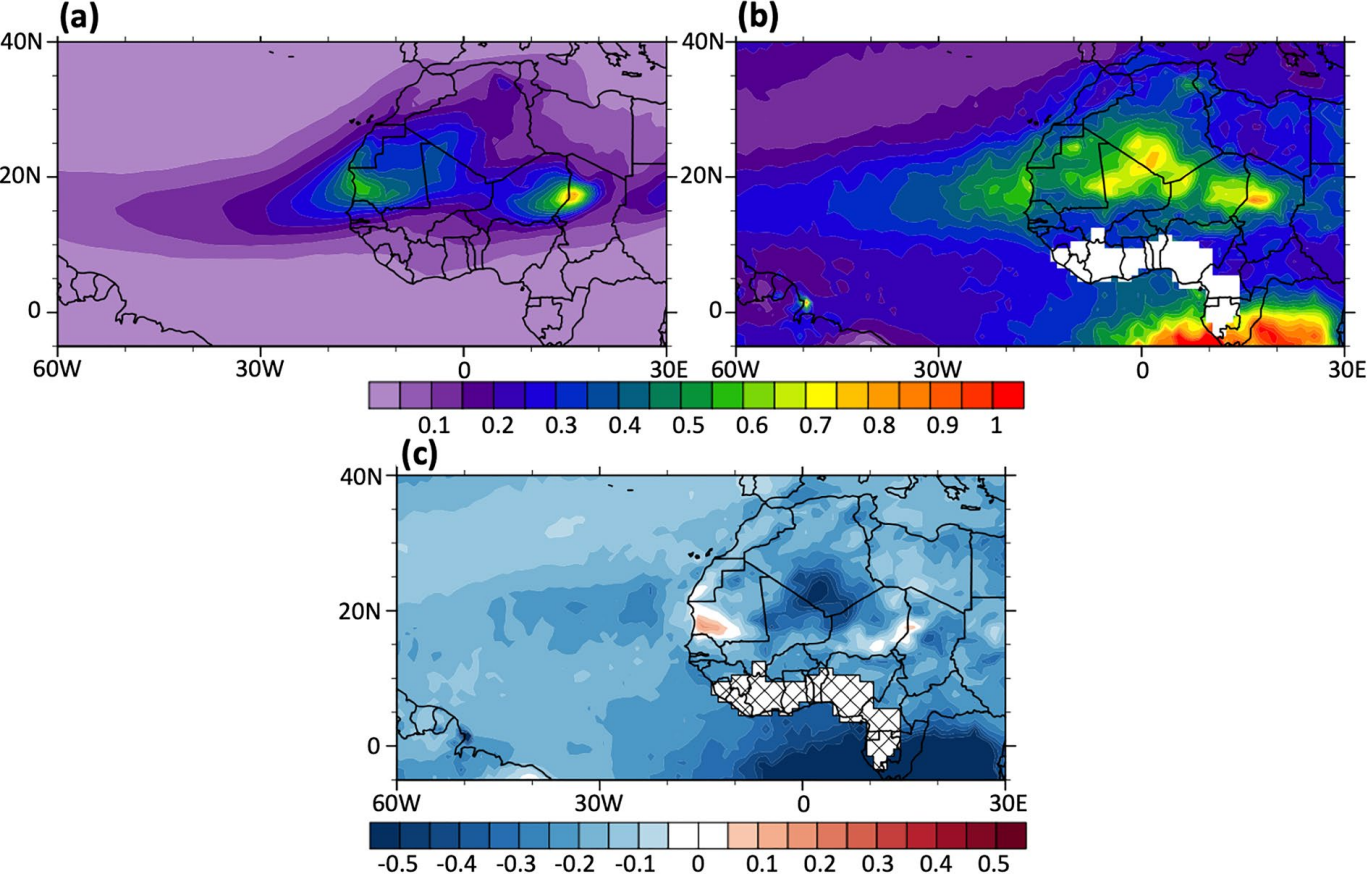
The 3‐month mean (July‐September, 2015) aerosol optical depth (AOD; unitless) at the wavelength of 550 nm from (a) DON simulation and (b) observation. (c) The AOD difference between (a) and (b) (DON—observation). The observed AOD are fused from the Moderate Resolution Imaging Spectroradiometer (MODIS) and the Multiangle Imaging Spectro Radiometer (MISR) instruments. MISR is on the Terra satellite; MODIS is on both the Aqua and Terra satellites.

We use the DON experiment result to evaluate the AWOD model performance, which includes both dust‐radiation and dust‐cloud interactions as in reality. Figure 1 shows the 3‐month mean aerosol optical depth (AOD) from satellite observation and DON at 12:00 UTC. The observed AOD is fused data measured by the Moderate Resolution Imaging Spectroradiometer (MODIS) instruments onboard Satellites Aqua and Terra and the Multiangle Imaging SpectroRadiometer (MISR) instrument onboard Satellite Terra. During the warm season, the AOD over North Africa and subtropical Atlantic is mainly contributed by dust. Since dust is the only aerosol type considered in the AWOD model, a relatively small AOD (about 0.1–0.15) contributed by marine aerosols (e.g., sea salt) is presented everywhere outside the dust plume over the ocean in observation, but not in the model result (Figure 1a versus 1b); in addition, the model misses the large AOD that is observed south of the equator between 10°W and 30°E due to biomass burning which was not included in the simulation. Nevertheless, although the model in general underestimates observed AOD values (Figure 1), both observations and model results share some similarities in AOD patterns, with high values located over central and western Saharan Desert extending to about 30°W and ridging at 15°N over the ocean following Saharan Air Layer (SAL) and AEJ (Chen et al., 2015; Karyampudi et al., 1999). The model nicely captures the maximum AOD (∼0.9) over the West Chad and East Niger (∼16°E and 17°N), which is one of the largest dust source regions over the Sahara Desert. However, the model misses the maximum AOD over southwestern Algeria and northeastern Mali (∼2°E and 23°N); this is mainly because the numerical simulation is a 4‐month free run and the model cannot accurately predict weather systems, which are responsible for dust emissions at the right locations with the right intensities (such as the Saharan heat low) over this region. The model also underestimates AOD (0.1–0.25) over central and western Atlantic after long‐range transport. This underestimation of long‐range transport can be attributed to errors from the dust parameterization (e.g., size and optical properties, and dust sedimentation) and the exclusion of sea salt in the model. Contrastingly, the observation has a smaller AOD value than modeled over western Mauritania (∼15°W and 17°N) and presents a discontinuous AOD feature crossing the coastline with a larger value over ocean and a smaller value over desert. This discontinuity is in part due to the use of different retrieval algorithms in observations between desert (deep blue algorithm; Sayer et al., 2013, 2014) and ocean (dark target algorithm; Remer et al., 2005).

As AOD is underestimated in DON, it will influence the evaluation of the dust‐related impacts on SST and air‐sea interactions, which is discussed in section 4. A sensitivity experiment that is identical to DON except for doubling the dust emission amount is performed, and its influence on AOD and SST is also discussed in Section 4.

**Figure 2:**
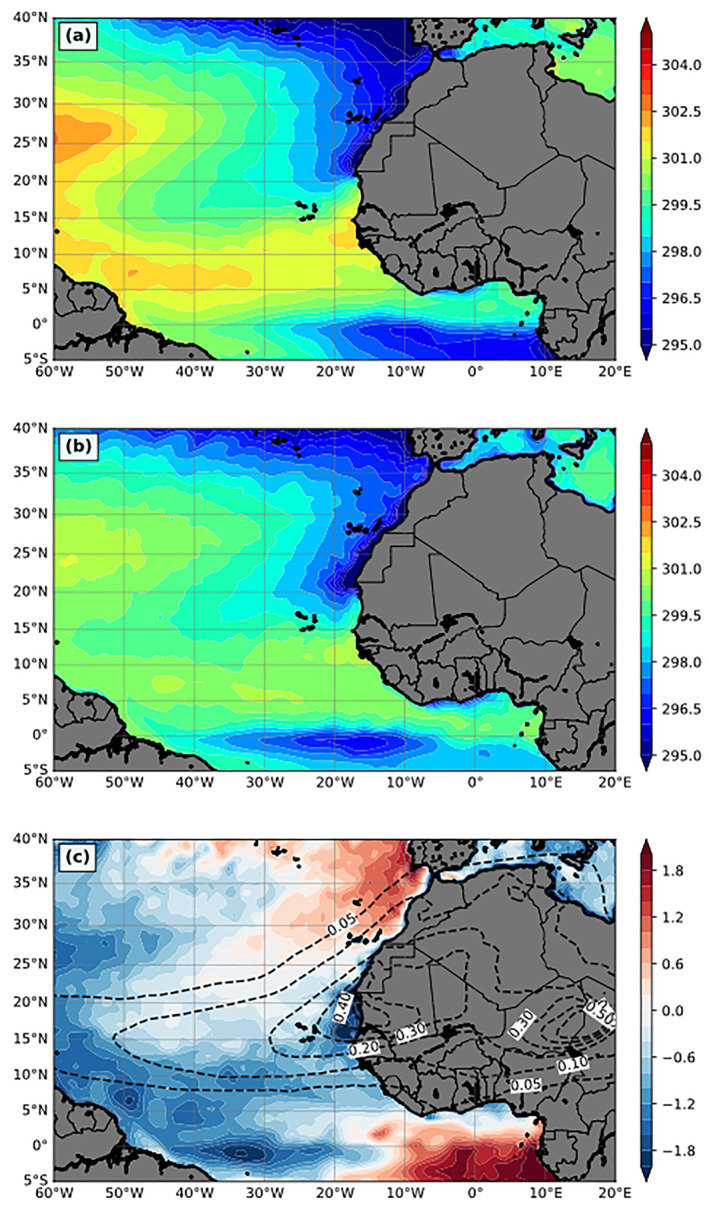
The 3‐month mean (July‐September, 2015) sea surface temperature (SST; K) from (a) CFSv2 analysis and (b) DON. (c) The difference between CFSv2 and DON (DON—CFSv2). The dashed contours in (c) are the AOD field from DON.

Figures 2a and 2b compare the 3‐month mean SST between CFSv2 and DON. The overall SST patterns are similar, including an SST warm ridge at about 10°N, the westward increase of SST north of 10°N over the Atlantic, and cold SST along the northwestern coast of North Africa due to coastal upwelling. However, the modeled SST field differs from the reanalysis in several regions. In particular, simulated SST values are about 2 °C cooler than the reanalysis over central to western Atlantic and along the 10°N temperature ridge, and 1–2 °C warmer over the Gulf of Guinea area and west of Morocco and Portugal (Figure 2c). The warm SST bias over the Gulf of Guinea area could be in part due to the neglect of biomass burning aerosols in the model (Figure 1c).

**Figure 3:**
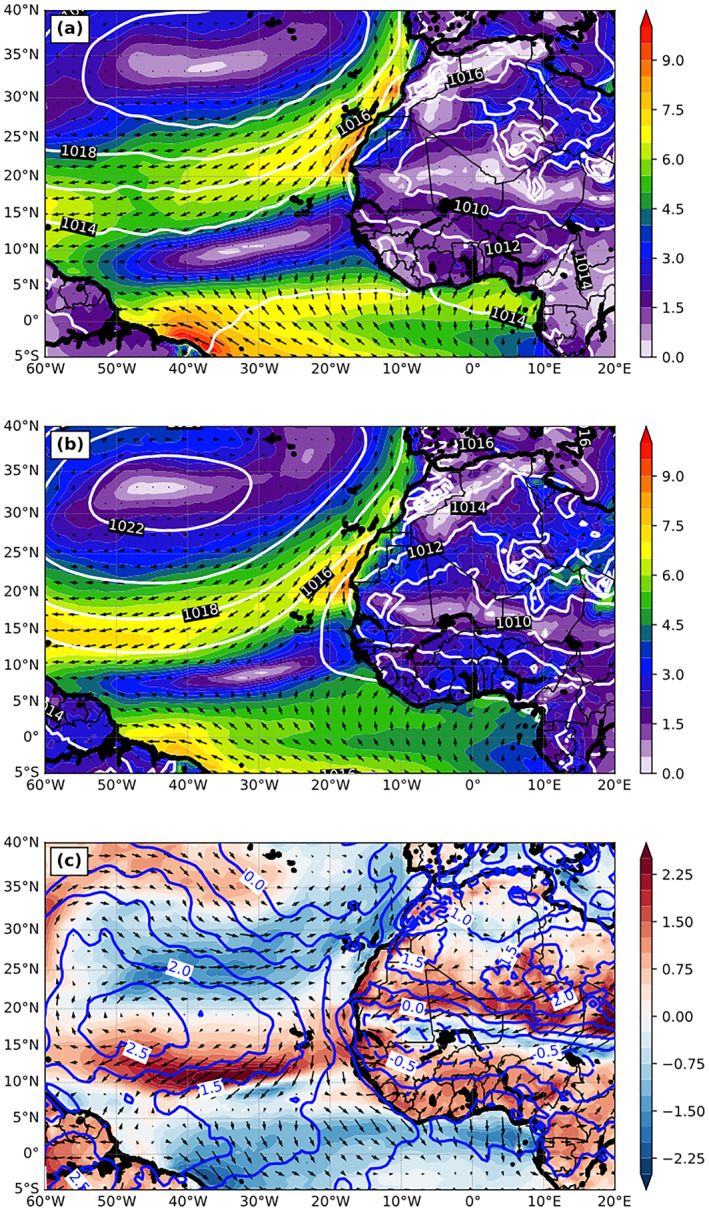
The magnitude of 3‐month mean (July‐September, 2015) 10‐m winds (m s^−1^; color shading) and sea level pressure (hPa; white contours) from (a) CFSv2 analysis and (b) DON, and (c) the difference of 10‐m winds (m s^−1^; color shading) and sea level pressure (hPa; blue contours) between CFSv2 and DON (DON—CFSv2).

In addition to the SDRF (to be discussed in the next section), many other meteorological fields can also affect SST and upper‐ocean dynamics, such as near‐surface wind, temperature, and moisture. Thus, we further evaluate these variables against analysis data (i.e., CFSv2). Figure 3 shows the magnitude of the 3‐month mean 10‐m winds (shading) and sea level pressure (SLP; contours). For 10‐m winds, both the model and analysis show a calm‐wind zone overlapping with the SST ridge at about 10°N, where convective clouds in the ITCZ are located. However, the analysis presents a wider calm‐wind zone in the meridional direction, which is consistent with a wider and warmer SST zone in the reanalysis. The modeled subtropical high is too strong and extends further south (e.g., the latitude of the 1,020 hPa isobar in Figure 3a versus 3b), resulting in weaker trade winds at 20–30°N and stronger trade winds at 10–20°N (i.e., shifted southward); moreover, the modeled southerly equatorial flow is too weak, forming a sandwich‐like pattern of wind anomaly over the ocean (Figure 3c). We note that the wind vector difference in Figure 3c can be contributed by the differences of both wind speed and direction.

**Figure 4:**
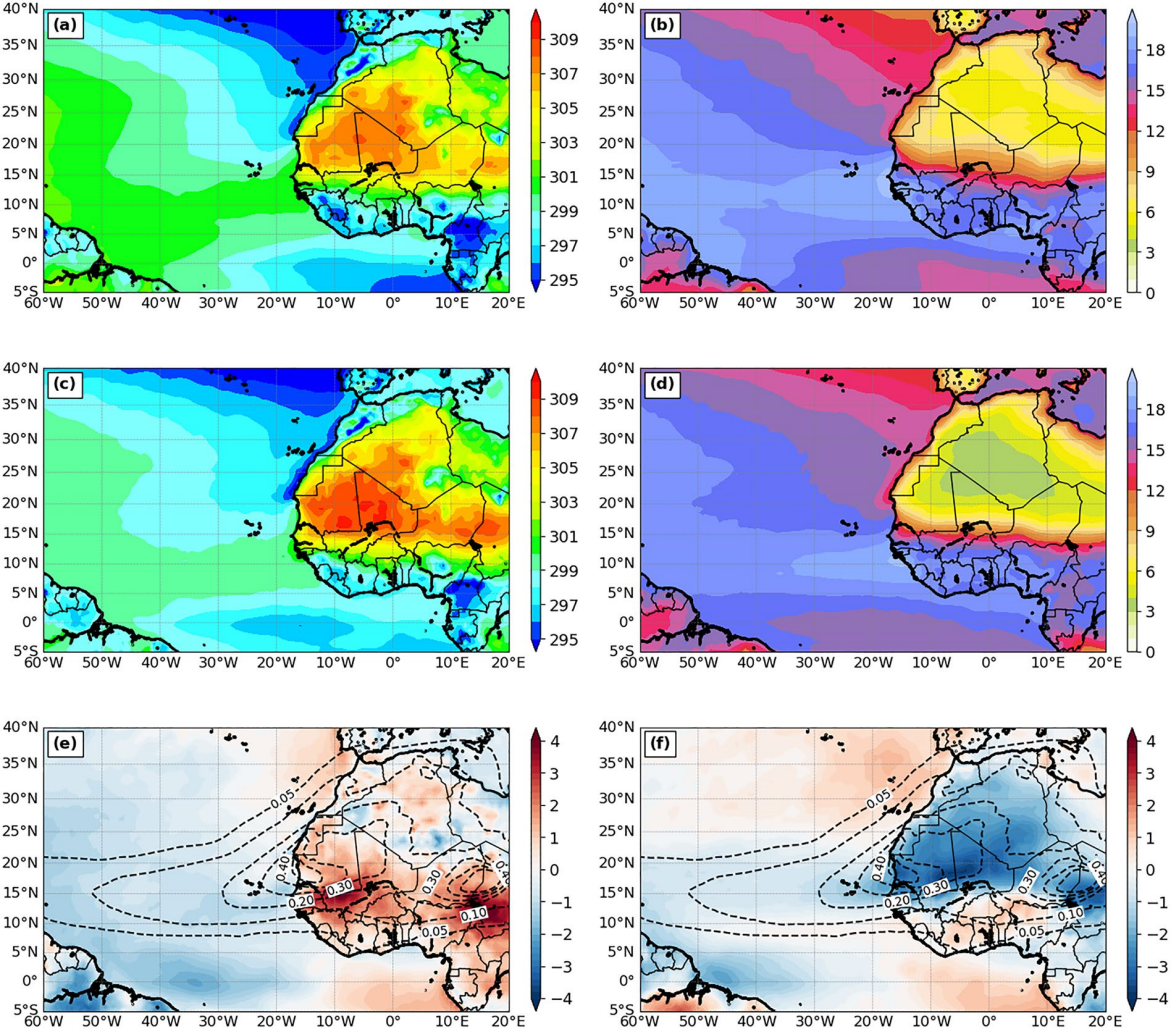
The 3‐month mean (July‐September, 2015) 2‐m temperature (K) from (a) CFSv2 analysis and (c) DON, and (e) the difference between CFSv2 and DON (DON—CFSv2). Panels in the right column are the same as those in the left column, except for 2‐m water vapor mixing ratio (g kg^−1^). The dashed contours in (e) and (f) are the AOD field from DON.

Figure 4 includes a comparison between reanalysis and modeled 2‐m temperature and moisture. As expected, the patterns in Figure 4 are similar to the SST pattern for both model and analysis, especially 2‐m air temperature, since these thermal fields are connected to each other through the surface energy fluxes. Both 2‐m temperature and moisture values increase toward the west. However, the modeled 2‐m temperature (moisture) is cooler (drier) than analysis over the western Atlantic and tropical North Atlantic regions.

In summary, compared with observations and analysis, some discrepancies exist in the modeled fields. However, most modeled patterns and values agree reasonably well, in particular considering that the model is freely integrated for four months, including 1‐month spin‐up time. Thus, this increases our confidence in the model's ability to reasonably simulate dust‐physical processes, which allows us to evaluate the impact of dust on the near‐surface meteorological variables, upper‐ocean processes, SST, and surface energy fluxes.

### 3.2 The Impact of Dust on Surface Downward Radiation Fluxes (SDRF)

**Figure 5:**
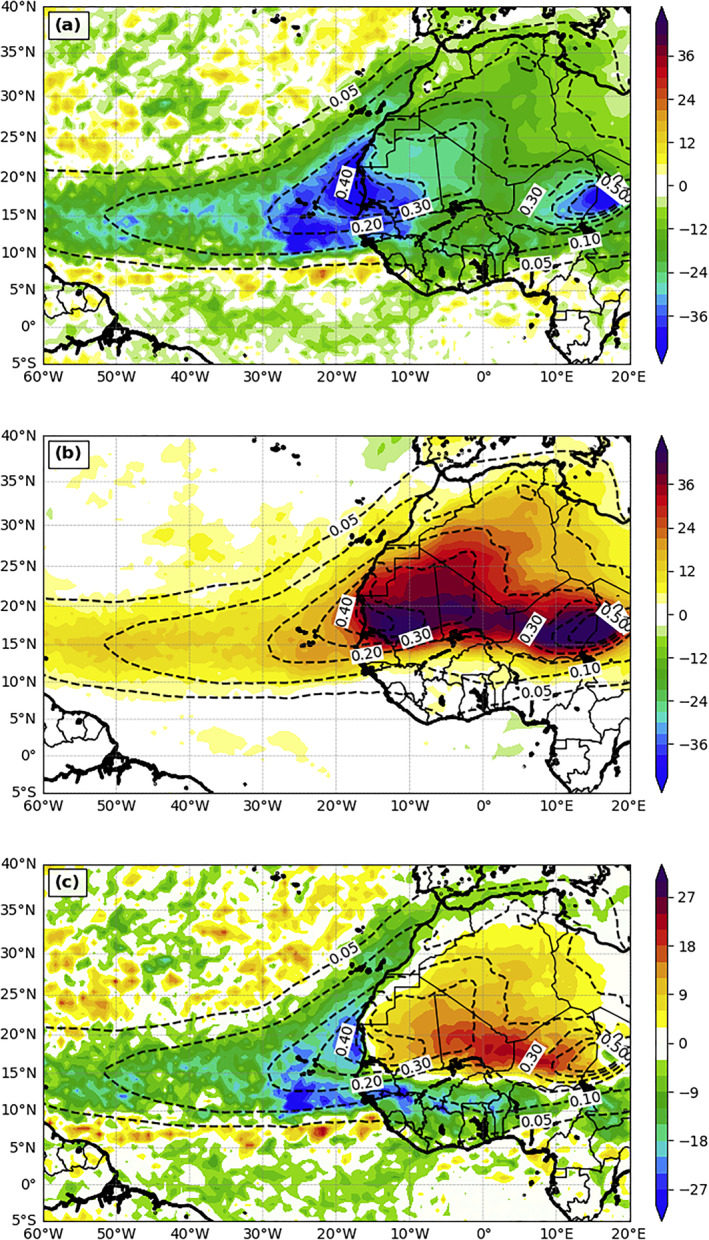
The differences of 3‐month mean (July‐September, 2015) (a) net surface downward shortwave radiation flux (W m^−2^), (b) downward longwave radiation flux at the ground (W m^−2^) between DON and DOFF (DON—DOFF), (c) is the summation of (a) and (b). The dashed contours are the AOD field from DON.

**Figure 6:**
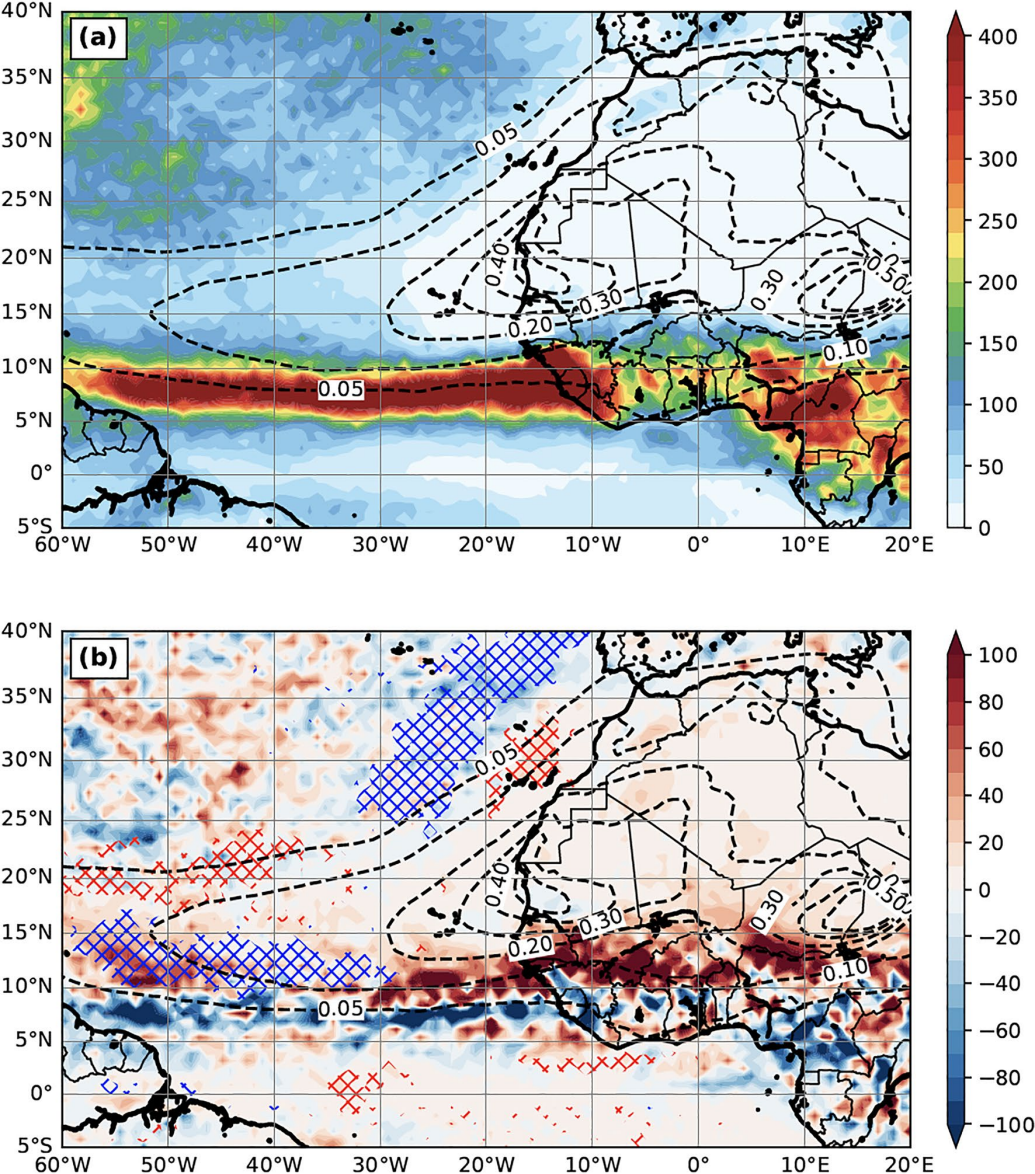
The 3‐month mean (July‐September, 2015) column integrated hydrometeors (g m^−2^) from (a) DOFF and (b) DON—DOFF. The blue (red) cross‐hatched lines in (b) indicate the differences of integrated hydrometeors (g m^−2^) of low‐level clouds, whose magnitudes are greater than 5 g m^−2^ with negative in blue and positive in red. The dashed contours are the AOD field from DON.

During the warm season, the main Saharan dust plume propagates from the desert to eastern Atlantic at about 30°W, and then reaches out to the central and western Atlantic with a much smaller concentration (represented as AOD in Figure 5). In general, the dust direct radiative effect attenuates the net downward SW radiation flux reaching the surface and increases the surface downward LW radiation flux. As presented in Figure 5a for the surface net downward SW flux and Figures 5b for the surface downward LW flux, similar patterns between AOD and the changes of SDRF, which is defined as the summation of the surface net downward SW flux and the surface downward LW flux, are observed. This dust‐radiation‐dominant scenario is mainly because the hot and dry SAL (10–25°N), where most of Saharan dust resides in summer, suppresses cloud development over the region (Figure 6a). The change in fluxes can reach −45 W m^−2^ for SW (Figure 5a) and 20 W m^−2^ for LW (Figure 5b) over the ocean. These magnitudes imply that the dust direct radiative forcing plays an important role in the changes of the surface radiation budget, in particular close to the East Atlantic. The combined changes of surface downward SW and LW fluxes (Figure 5c) indicate that the dust‐LW effect dominates over the source region (e.g., desert) and the dust‐SW effect dominates over the remaining of the dust coverage areas. For a 3‐month average, the magnitude change of the combined SDRF can reach as high as 30 W m^−2^ over the ocean (Figure 5c), which is quantitatively similar to those obtained in Strong et al. (2015) and Jordan et al. (2018).

While the patterns of AOD and SDRF changes are similar, discrepancies between these patterns still exist (Figure 5), which are attributed to cloud changes by the dust indirect and semidirect effects. Figure 6a shows the main cloudy areas over the domain of interest, including the ITCZ and southern North Africa. Outside the major dust plume region, and in particular over the southern edge of the dust plume, the increase and decrease of SDRF by dust both match the decrease and increase, respectively, of cloud amounts immediately above quite well over the ocean (Figure 5c versus Figure 6b). Furthermore, the changes of the SDRF over these regions (Figure 5c) are primarily attributed to the cloud‐SW interaction (Figure 5a). For example, dust‐cloud effects increase the surface downward SW radiation flux at the southern edge of the dust plume (∼8°N) and this is due to the slightly northward shift of ITCZ. Similarly, the reduction of the surface downward SW flux (∼10–15°N between 0°W and 30°W) immediately north of the increased radiation flux band is due to the increase and northward shift of clouds. We note that there is a positive SDRF anomaly north of the dust plume between 15°W and 35°W and this is attributed to both cloud‐SW and cloud‐LW effects caused by the dust‐induced reduction of low‐level clouds (LLCs) (blue cross‐hatched lines in Figure 6b). Here we define LLCs when hydrometeors exist between the surface and 680 hPa but not between 680 and 440 hPa (Rossow & Schiffer, 1999).

### 3.3 The Impact of Dust on the Air‐Sea Interface and Upper‐Ocean Characteristics

In this subsection, we examine the impact of dust on the near‐surface fields and processes, such as near‐surface meteorological variables, WSC, and dynamical processes within the upper‐ocean layer, by comparing results from DON and DOFF experiments. The changes of these variables and processes will be further used to interpret the changes of SST and surface LHF/SHF by dust in section 4. Below we begin with near‐surface meteorological variables, which are the main drivers of upper‐ocean thermodynamics and dynamics.

#### 10‐m Wind and 2‐m Temperature and Moisture

**Figure 7:**
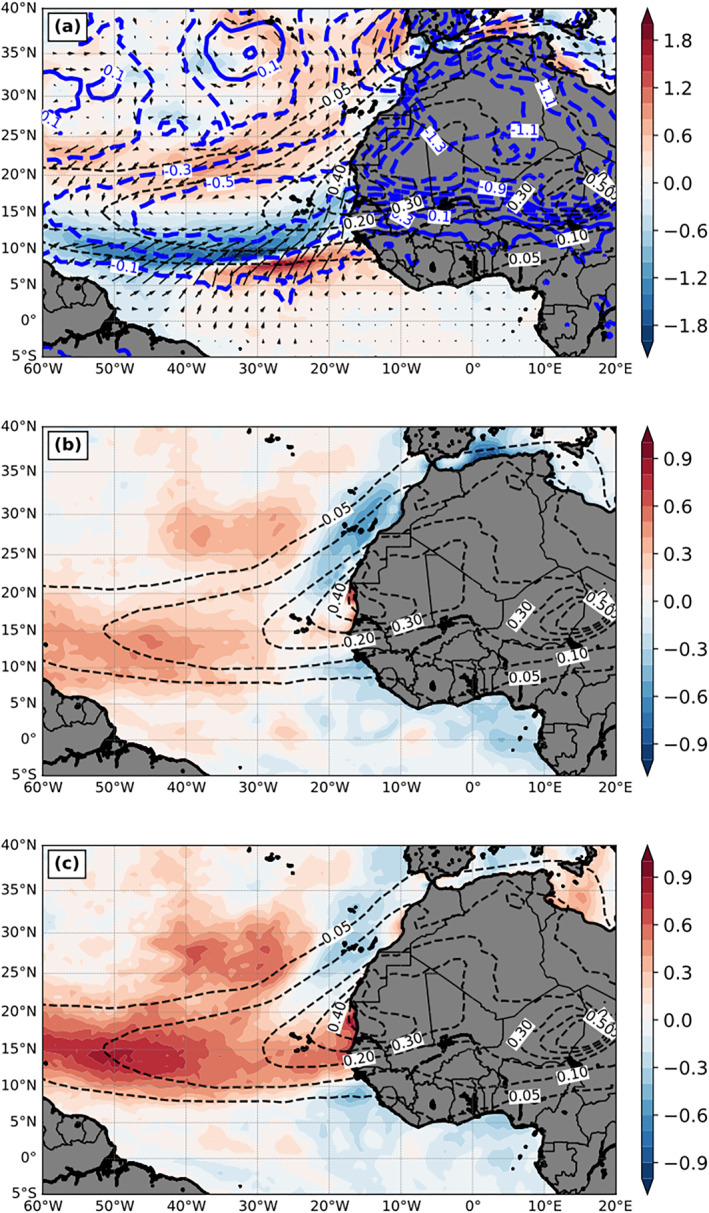
The differences of 3‐month mean (July‐September, 2015) (a) 10‐m wind speed (m s^−1^) and vectors and sea level pressure (hPa, blue contours), (b) 2‐m temperature (K), and (c) 2‐m water vapor mixing ratio (g kg^−1^) between DON and DOFF (DON—DOFF). The black dashed contours are the AOD field from DON.

Figure 7a shows dust‐induced 10‐m wind and SLP changes. Dust causes negative SLP anomalies with a change of roughly −0.5 hPa over ocean, similar to that in Jordan et al. (2018), and approximately −1 hPa over land underneath the dust area due to dust radiative heating in the atmosphere. This negative pressure anomaly drives a mean cyclonic circulation anomaly. Over the ocean, the changes of 10‐m winds by dust (e.g., a sandwich‐like pattern) include (1) the intensification of the eastern and southern branches of the anticyclonic circulation associated with the subtropical high, (2) the weakening of southeasterly cross‐equatorial flow (i.e., southwesterly anomalies) over the northwestern tropical region, and (3) the intensification of southwesterly cross‐equatorial flow southwest of West Africa (Figures 3b and 7a). The wind changes over the southern edge of the dust plume partially reflect the northward shift of ITCZ by dust, as discussed earlier. It is interesting to see that over the ocean the sandwich‐like pattern of 10‐m wind changes induced by dust (Figure 7a) reduces part of the 10‐m wind bias in DON against analysis (i.e., opposite signs between Figures 7a and 3c), but shifts slightly southward. Similarly, the dust‐induced SLP change reduces the SLP bias in DON as shown in the same figures. Thus, the inclusion of the dust‐physical processes in AWOD simulation reduces the 10‐m wind and SLP biases over the tropical and subtropical North Atlantic Ocean.

Figures 7b and 7c show the dust‐induced 2‐m temperature and moisture (mixing ratio) changes, respectively, over the ocean; their patterns are quite similar. Dust decreases 2‐m temperature (minimum about −0.6 K) and moisture (minimum about −0.6 g kg^−1^) surrounding the coastal region of North Africa, except around 15°N, where dust increases both fields from the western African coast toward west along the major dust plume region. Dust also increases 2‐m temperature and moisture north of the dust plume around 23–32°N and 25–45°W. The changes by dust range from −0.6 to 0.6 K for temperature and from −0.6 to 0.9 g kg^−1^ for moisture, with the maximum positive changes west of 40°W. These patterns are similar to but are not quite the same as SST changes by dust that will be discussed in Section 4. The similarity of their patterns is again because these three variables (2‐m temperature and moisture, and SST) interact closely with each other through surface energy fluxes (i.e., air‐sea interactions).

#### Wind Stress Curl (WSC), Sea Surface Height (SSH), and Upper‐Ocean Mixed Layer and Current

Surface wind stress –$\tau \;(=\tau_{x}\hat{i}+\tau_{y\;}\hat{j}$) where, $\tau_{x}$ and $\tau_{y}$ are the east‐west and south‐north components, respectively—has the same direction as near‐surface wind, while the magnitude is proportional to the square of the difference between 10‐m wind and ocean‐surface current (Fairall et al., 1996). Because of the Earth's rotation, the wind stress induces a rightward water mass flux within the upper‐ocean layer in the northern hemisphere. Thus, positive (negative) vertical component of WSC, i.e., $(\;\frac{\partial \tau_{y}}{\partial x}-\frac{\partial \tau_{x}}{\partial y})\;\hat{k}$, will induce mass divergence (convergence) in the upper‐ocean layer, locally reducing (raising) the SSH and shallowing (deepening) the mixed layer due to the upwelling (downwelling) water. The upwelling (downwelling) can generally cause cold (warm) SST response.

**Figure 8:**
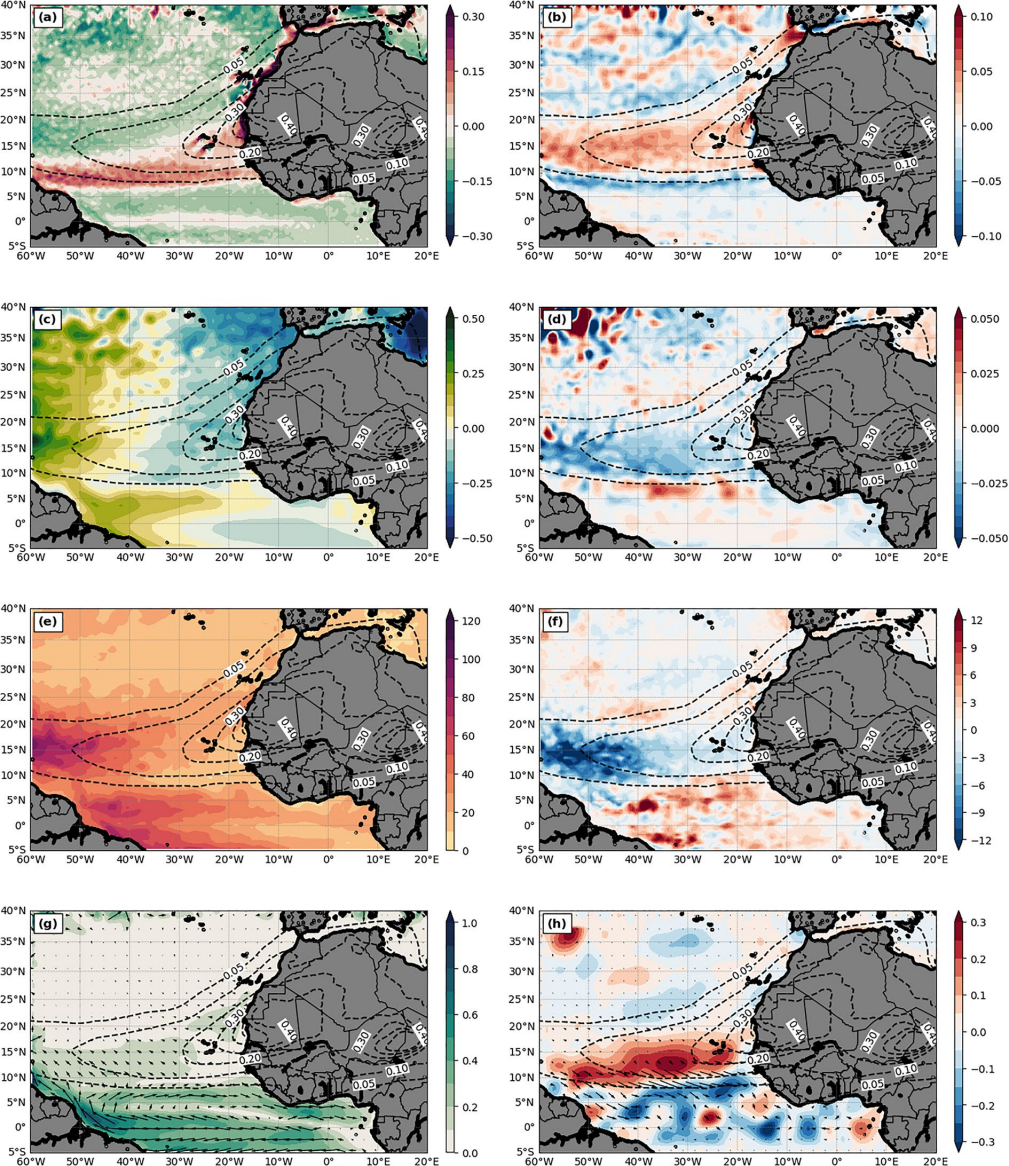
The 3‐month mean (July‐September, 2015) wind stress curl (WSC; 10^−6^ N m^−3^) for (a) DOFF and (b) DON—DOFF. (c) and (d) are the same as (a) and (b), respectively, except for the sea surface height (SSH; m). (e) and (f) are the same as (a) and (b), respectively, except for the mixed layer depth (MLD; m). (g) is upper‐ocean 10‐m current (m s^−1^) for DOFF and (h) is upper‐ocean 70‐m divergence difference (shading; ×10^−6^ 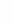
s^−1^) and 10‐m current difference (vectors; m s^‐1^) between DON and DOFF (DON—DOFF). The dashed contours are the AOD field from DON. Spatial filter is employed in (h) to remove the impacts of tropical instability waves.

Figure 8a shows the 3‐month mean WSC from the DOFF experiment, whose pattern is consistent with the curl of near‐surface wind (see Figure 3b from DON 10‐m wind as a reference) and the magnitude of WSC over Atlantic is about 3 × 10^−7^ N m^−3^. Since dust greatly modifies the near‐surface wind (Figure 7a), it can alternatively change the WSC (Figure 8b) and importantly, the change of the magnitude is comparable to the WSC magnitude itself over some regions. The dust‐induced near‐surface cyclonic circulation anomaly (i.e., due to negative SLP anomaly) centered at 15°N produces a positive WSC anomaly. To its north, the intensification of the subtropical high circulation by dust at its south and southeast sectors produces a negative WSC anomaly (∼22–27°N). To the north of this negative WSC anomaly, dust produces a narrow‐banded positive WSC anomaly (∼30°N) due to 10‐m easterly wind anomaly to its north (i.e., the positive pressure anomaly) as shown in Figure 7a. Along the 8°N where ITCZ is located, the negative WSC difference is produced due to the intensification of cross‐equator northward flow (Figure 7a) after the inclusion of dust‐physical processes.

The 3‐month mean SSH is generally higher over western Atlantic as shown in Figure 8c due to the typical western intensification and the recirculation associated with the western boundary current in the North Atlantic. In terms of the dust impacts, part of the SSH changes by dust (Figure 8d) corresponds relatively well to the change of WSC (Figure 8b), i.e., negatively correlated as discussed earlier. At 8°N, the negative WSC anomaly, which induces convergence, increases the SSH. On the other hand, the positive WSC anomaly, which induces divergence centered around 15°N, decreases the SSH. These results confirm the large‐scale change of the SSH due to the WSC change by dust, though the negative correlation almost disappears north of 20°N.

Figure 8e shows 3‐month mean upper‐ocean mixed layer depth (MLD) from DOFF. A deeper upper‐ocean mixed layer is presented over western Atlantic between 10°N and 25°N resulting from the subtropical gyre recirculation. The change of the MLD, where the temperature is relatively uniform, can largely modulate the SST change. The shallower a mixed layer is, the more the layer can be warmed up with a fixed energy input, thus easily increasing the SST if the entrainment at the bottom of the mixed layer is small. Figure 8f shows the MLD change by dust. The most prominent feature is the decrease of MLD around 15°N, which is primarily due to the positive WSC anomaly change by dust over the region (Figure 8f). However, the coverage of negative MLD change is larger than that of positive WSC change, such as off coastal areas of northeastern Guyana, Suriname and French Guiana. This implies that other mechanisms are also involved in the change of MLD, further addressed in the next section.

Lastly, the upper 10‐m current shows relatively weak upper‐ocean flow, except north equatorial current (10–15°N) and equatorial current system (Figure 8g), e.g., the south equatorial current near the equator and the north equatorial counter current (NECC) at 3–10°N. The current speed is shaded, highlighting the strength of these currents. The dust‐physical processes modify upper‐ocean circulation, which further changes the upper 70‐m divergence (consistent with 10‐m current difference between DON and DOFF), as shown in Figure 8h. Since the ocean divergence field and 10‐m current differences are usually noisier due to the prominent mesoscale features in the high‐resolution simulation, a spatial 300‐km filter is employed to highlight the large‐scale differences. Dust causes divergence underneath the dust plume, which is consistent with the positive WSC change over the region. Similarly, to the south of the dust plume (approximately south of 10°N), the convergence dominates due to the negative WSC change (Figure 8b), leading to the increase of MLD in DON (Figure 8f).

## 4 Dust Impact on SST Changes and the Surface Energy Exchanges

As mentioned earlier, SST impacts the ocean‐atmosphere energy exchange, specifically LHF and SHF, and can be modulated by dust through several thermodynamics and dynamical processes. Keeping this in mind, several questions are raised: “Does the modification of SDRF by dust directly correlate to SST change?” “If the answer is no, what are other dust‐induced processes that could be responsible for SST change?” Furthermore, surface winds are also modified by dust‐physical processes and may impact the surface energy exchange. Thus, another question to ask is “In addition to SST change, which are the dust‐induced processes that could be attributed to LHF and SHF changes?” These questions are addressed in this section, in which we first compare the dust plume shape with dust‐induced SST, LHF and SHF change patterns and then discuss the mechanisms responsible for these changes.

### Patterns

**Figure 9:**
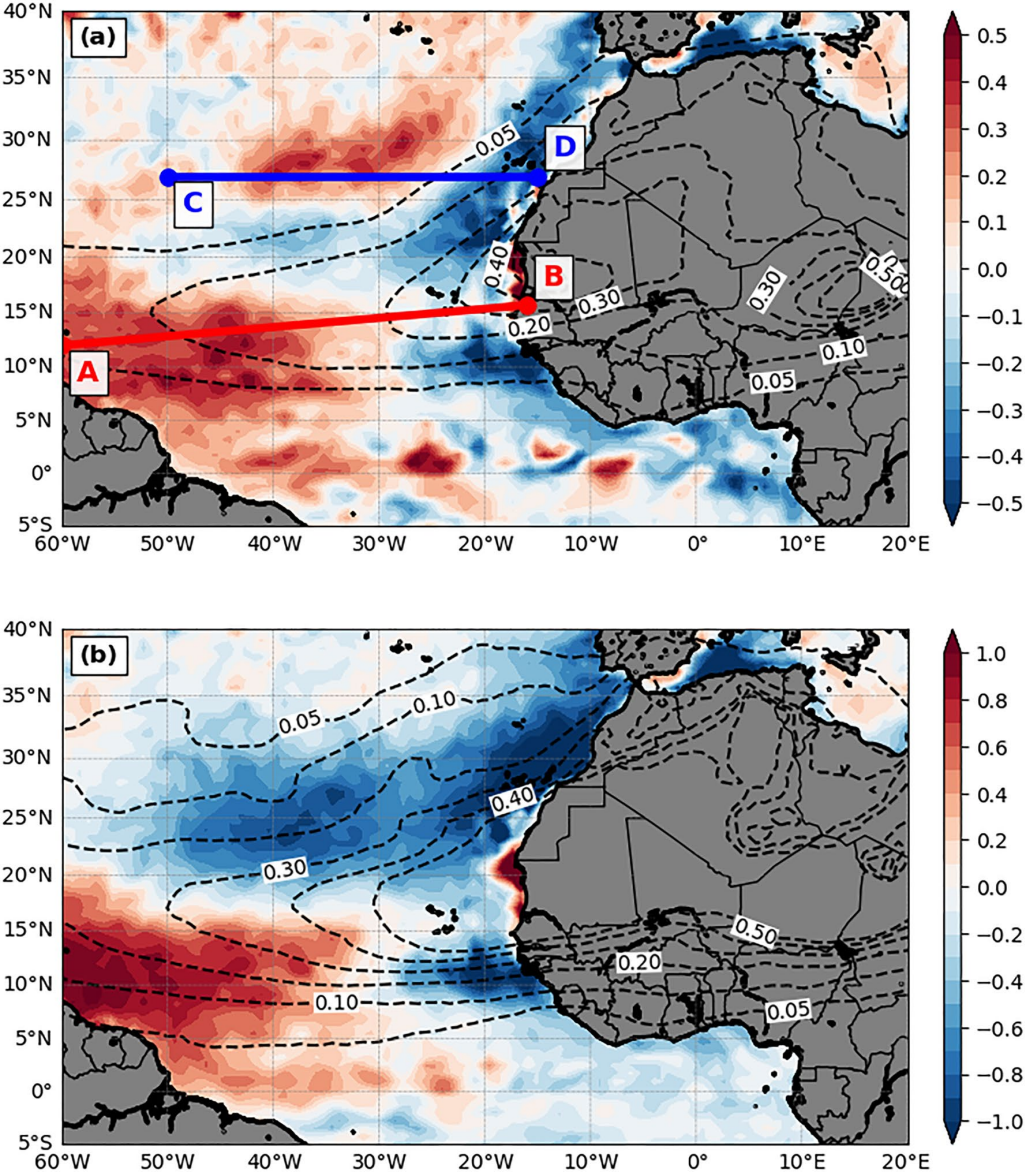
(a) The differences of 3‐month mean (July‐September, 2015) SST (K; color shading) between DON and DOFF (DON—DOFF). The dashed contours are the AOD field from DON. The red line AB and blue line CD indicate the locations of the vertical cross section in Figure 11. (b) same as (a) but for the doubled dust emission run relative to the DOFF.

Figure 9a shows the 3‐month mean SST changes by dust (i.e., DON‐DOFF). The impacts of dust on SST changes range from −0.5 to 0.5 K. Surrounding the West African coast, the presence of dust mostly lowers SST values, except near the maximum dust plume region. Among these cold SST anomalies, colder values are located underneath the dust plume (i.e., west‐southwestern, west‐northwestern, northwestern, and northern sides of the coast) as expected. However, the cooling extends westward along the northern (∼22°N) and southern (∼11°N) edges of the dust plume with an asymmetric feature, in which the southern branch extends to 30°W and the northern branch extends farther west until 55°W. In contrast to SST cooling, which is more intuitive, dust unexpectedly warms the ocean‐surface at several other regions, such as the large subtropical area (0–20°N and 30–60°W) and the northeast‐southwest elongated zone north of the major dust plume between 25°N and 35°N. The warming within the subtropical area also shows an asymmetric feature with respect to the dust plume (e.g., maximum SST warming located in the southern side of the plume).

A sensitivity experiment with doubled dust emission is performed. The simulated AOD is higher (Figure 9b), as expected, and the magnitude is more than twice of the value in DON (Figure 9a) due to nonlinear effects; for example, more dust can cause stronger radiative heating (i.e., higher buoyancy) at the upper dust plume, which will bring more dust to a higher elevation (figure not shown) and remain in the atmosphere for a longer time. The SST difference pattern caused by doubled dust emission is similar to that in DON with a larger magnitude of difference (Figure 9a versus Figure 9b), except that the SST warming north of 25°N in DON becomes a SST cooling anomaly over the region after doubling the dust emission. This pattern is expected and occurs mainly because dust with a larger emission amount is able to extend further north and reduces the SDRF over the region.

The unexpected SST warming by dust in our study is very different from those in Strong et al. (2015) and Jordan et al. (2018), who used a coarse‐resolution coupled global model for long‐term simulations. In these previous studies, dust cools SST over the entire tropical and subtropical North Atlantic except the Gulf of Guinea. Two possible differences between their simulations and ours could potentially explain such dust‐induced SST difference divergence, in addition to the different numerical models and physics schemes used. One is the much coarser resolution used in these previous studies (around 2° in their studies versus 15 km in ours), which cannot fully resolve many oceanic features, such as tropical instability waves, ocean mesoscale eddies and coastal upwelling. These features have been shown to be important to affect the ocean‐atmosphere interaction (Seo & Xie, 2011; Small et al., 2008, 2014; Tubul et al., 2015; Wu & Bowman, 2007). The other is the different simulation period (150–1,800 years in their studies versus one season in this study). The longer‐term accumulation of the negative dust radiative feedback on SST changes might extend the cooling difference further in space.

To explore the first difference, additional coarse‐resolution sensitivity experiments (approximately 60 km resolution), otherwise the same as DON and DOFF, are performed. The SST difference pattern with a coarse‐resolution differs from that with a higher resolution in Figure 9a. Besides expected weaker and smoother SST anomalies, the coarser‐resolution simulations significantly limit northern branch SST cooling extending westward (figures not shown). In addition to the impacts of unresolved mesoscale ocean eddies, this is in part due to that much less dust is emitted from the Sahara Desert since a 60 km horizontal spatial resolution cannot properly resolve those weather systems that uplift dust. We note that dust was prescribed in the simulations of Strong et al. (2015) and Jordan et al. (2018). Our sensitivity test results are more similar to Lau et al. (2009), who also suggested the warming of SST (personal communication) and 2‐m temperature by dust over the western Atlantic and Caribbean. They conducted eight seasonal simulations (April to October) using a coupled atmosphere‐ocean global model with 2 ×_ _2.5° resolution, though a simple mixed layer ocean model was used in their study. This similarity implies that longer‐term extension of the radiative cooling effect in Strong et al. (2015) and Jordan et al. (2018) might be the primary cause of the SST differences by dust between their investigations and ours.

**Figure 10:**
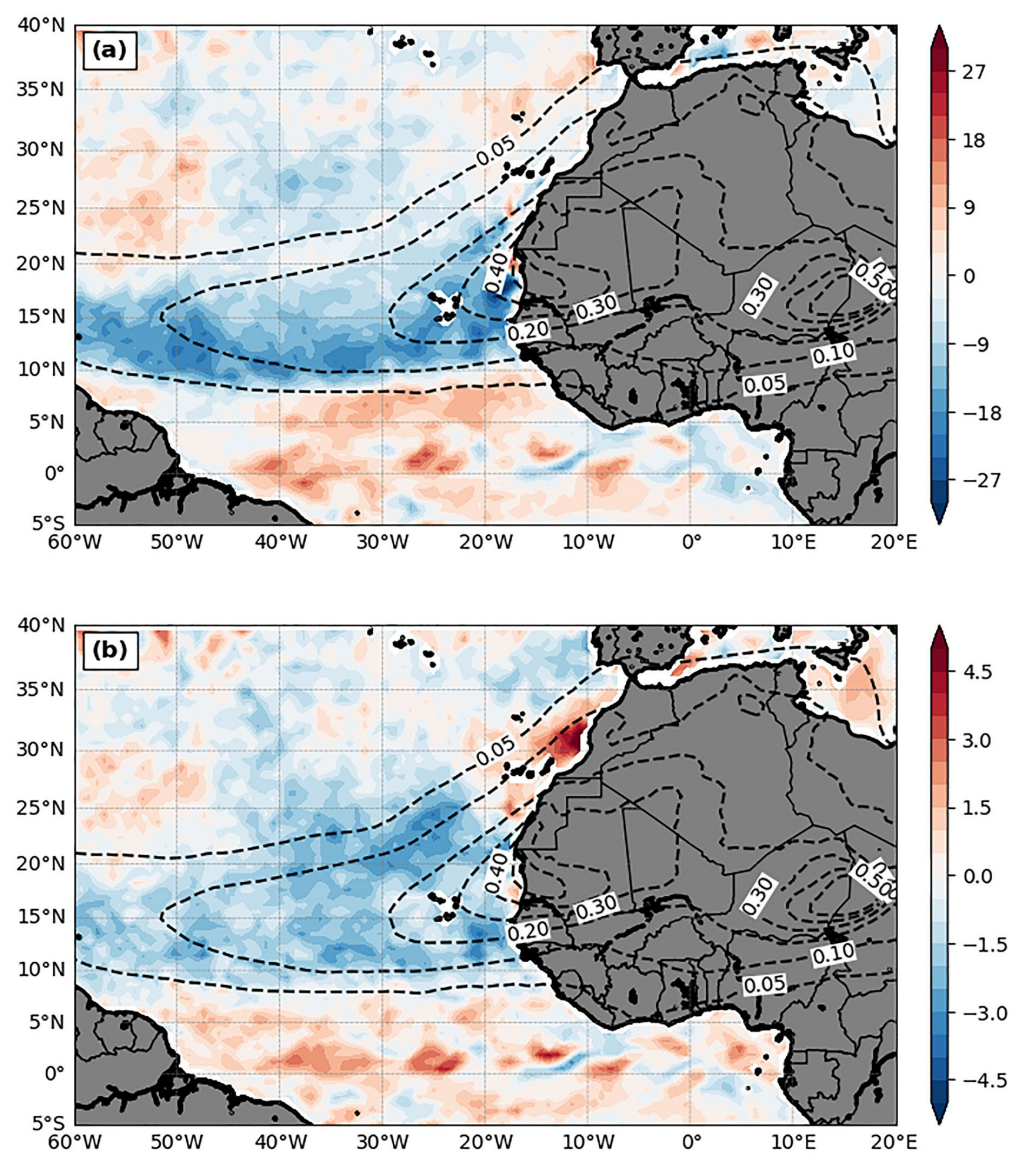
Same as Figure 9a but for (a) LHF (W m^−2^; color shading) and (b) SHF (W m^−2^; color shading), respectively.

Figures 10a and 10b show 3‐month mean LHF and SHF differences between DON and DOFF. Similar to their full fields (figure not shown), the LHF difference is much larger than the SHF difference as expected (positive upward). The patterns of dust‐induced LHF and SHF changes are quite similar, but are significantly different from that of the SST changes. Interestingly, while the dust plume shape is very different from the pattern of SST changes, it is more consistent with changes to LHF and SHF fields. Both upward sensible and latent energy fluxes are reduced by dust underneath the main plume region. However, a more careful comparison reveals that their flux reduction patterns are different in detail. These regional differences play some roles in the SST changes which cannot be well explored using the coarser‐resolution global model (Jordan et al., 2018; Strong et al. 2015). The negative LHF change has the maximum magnitude over the southern portions of the dust plume, while the negative SHF change is more evenly distributed under the entire dust plume. Additionally, over the offshore region of northwestern North Africa (between 22°N and 35°N), positive SHF changes can be observed just outside the coast underneath the dust plume (Figure 10b) and positive LHF changes are located further away from the coast and extend beyond the coverage of dust plume (between 30°N and 40°N). The influence of dust on both LHF and SHF is not limited underneath the major dust plume region. Both flux changes in general are positive to the south of the dust plume and the wave‐like pattern immediately north of the equator suggests the clear signature of tropical instability waves.

### Mechanisms

As discussed earlier, only part of the dust coverage area coincides with SST cooling anomalies, and the resultant cooling/warming feature is asymmetric with respect to the dust plume. This implies that SST changes by dust are not solely controlled by the change of SDRF (dust radiative effects), but also by other mechanisms such as those discussed in section 3.3, which will be addressed here.

Partially attributed to the reduction of SDRF (Figure 5c), the cold SST anomaly off coastal areas along northwestern North Africa between 15°N and 35°N can be explained further by the enhancement of upwelling due to the dust‐induced northeasterly anomaly in DON (red shading in Figure 7a). Also, the cold SST anomaly offshore of the southwestern North Africa or the southern edge of the dust plume around 10°N (Figure 9a) can be further explained by the intensified northward to northeastward winds in DON (Figure 7a), which enhances the LHF release to the atmosphere (i.e., positive LHF difference in Figure 10a).

**Figure 11:**
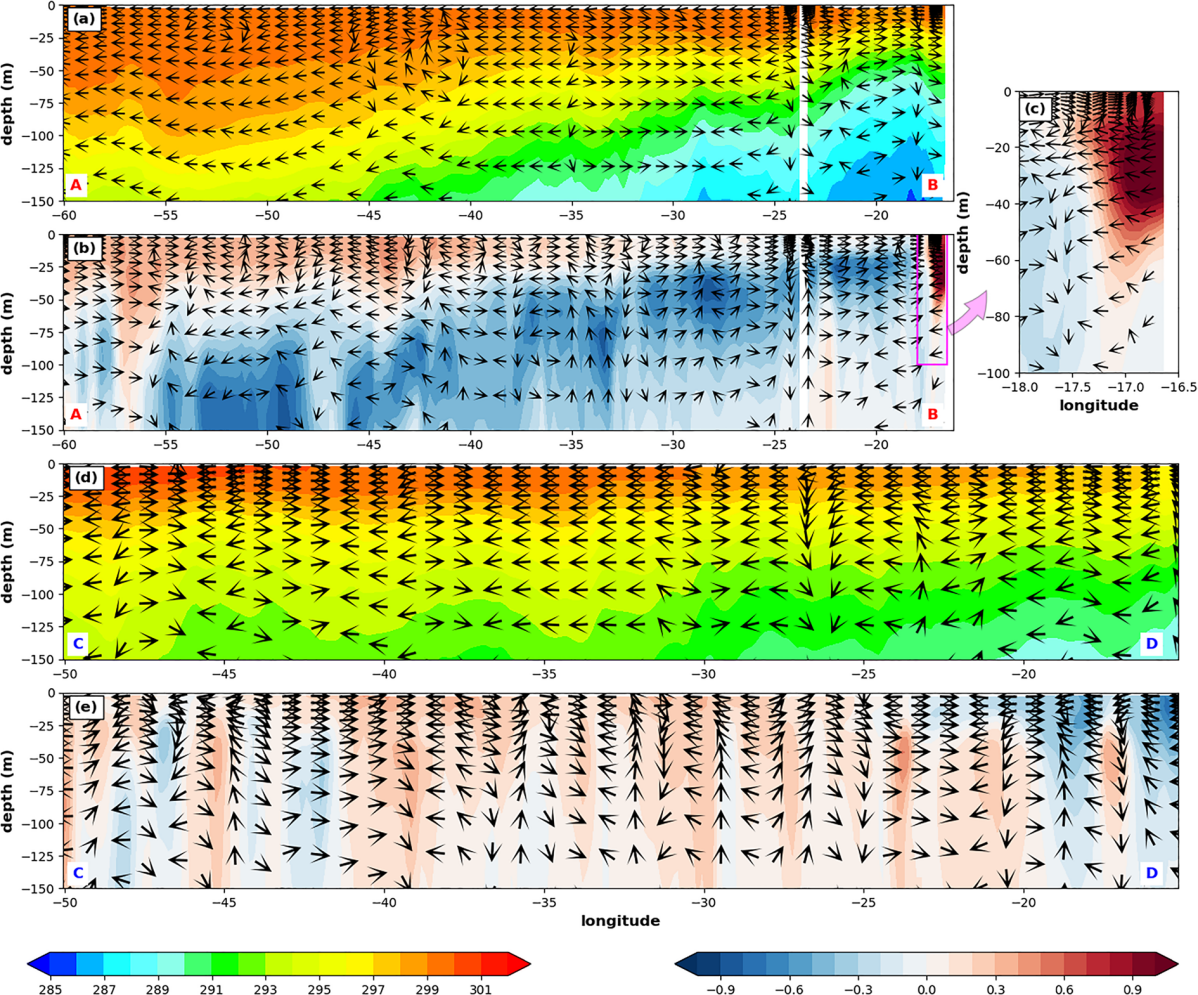
The vertical cross section of 3‐month mean (July‐September, 2015) ocean current (arrows; the projection of horizontal current and 10,000 times of vertical current over the vertical cross section), and temperature (K; color shading) from (a) DOFF and (b) DON—DOFF along the red line AB in Figure 9a. (c) is the enlargement of the magenta box in (b). (d) and (e) are the same as (a) and (b), respectively, but along the blue line CD in Figure 9a.

Along 15°N close to the coast of West Africa where the dust maximum locates, the reduction of SDRF is almost compensated by the warming effect due to the decrease of MLD by dust (Figure 8f), resulting in almost no SST change. This is also consistent with the inshore (near surface) and downwelling velocity vector difference (Figure 11c) and echoed by the cold temperature anomaly near the thermocline region (i.e., decrease of MLD) east of 30°W shown in the Figure 11b (line AB in Figure 9a). Figure 11a shows the vertical cross section of ocean temperature in the upper 150‐m depth from DOFF for reference. We emphasize that these reduced upwelling and compensation features have spatial scales within one degree along the coast and thus cannot be well‐represented by any coarse‐resolution global coupled model (e.g., Jordan et al., 2018). By contrast, our high‐resolution regional coupled model provides more detailed ocean dynamical processes.

The unexpectedly large warm SST anomaly region over the southwestern part of the dust plume (∼30–60°W and 8–20°N in Figure 9a) is attributed to two mechanisms, which supersede the SST cooling due to the reduction of SDRF by dust. First, a significant shallower MLD (Figure 8f) by dust‐induced positive WSC (Figure 8b) over the region can result in a warmer mixed layer (i.e., easier to heat up the mixed layer; Figure 11b) so as to SST. Second, weaker surface winds by dust (Figure 7a) can further reduce LHF and SHF (Figures 10a and 10b) with resulting increases to SST. To further demonstrate these processes, we examine upper‐ocean temperature and current differences between DON and DOFF passing through this region (line AB in Figure 9a). The warming near the surface and cooling below (Figure 11b) is consistent with the decrease of the MLD, which is due to increased WSC (i.e., induced divergence at upper 70 m in Figure 8h) and weakened trade winds by dust. The weaker trade winds reduce the vertical mixing in the ocean (i.e., stronger stratification), causing the warm (cold) anomaly in the upper (lower) layer in the DON. This increase of SST with the negative (i.e., reduced) LHF/SHF suggests that the surface wind change by dust plays an important role in the air‐sea flux change in this region (Wu et al., 2006). It is noticed that the warmer SST covers a larger area than that of the shallower MLD and weaker trade winds over this region. Specifically, there is a warm SST anomaly south of the dust plume and west of 40°W. The dust‐induced 10‐m southwesterly anomaly over this region can induce southeastward current anomalies near the ocean‐surface (Figure 8h), which advect warm water to this region. The dust‐induced SST warming north of the equator between 30°W and 40°W might result from the ocean current convergence change in the tropics (Figure 8h) which reduce equatorial upwelling. Note that as the dust‐induced change of the current convergence is relatively nosier compared to other mechanisms, a smoothing filter was applied to the change field for proper comparison.

The SST warming near the equator exhibits a wave‐like pattern. The warming west of 20°W roughly corresponds to a large patch of upper‐ocean convergence (i.e., negative divergence in Figure 8h), suggesting the important role of ocean currents in influencing SST values. The wave‐like SST differences may correspond to the different phase speed/occurrence of the tropical instability waves generated in the tropical Atlantic in these two cases. A long‐term model comparison study can help to further clarify this and is beyond the scope of this study.

Lastly, the dust‐induced large warm SST difference north of the dust plume (25–35°N) between 20°W and 43°W is due to the increase of SDRF (Figure 5c) caused by the reduction of LLCs by dust (Figure 6b) and the decrease of MLD caused by the increase of WSC (∼30°N) south of the enhanced subtropical high by dust (Figures 7a and 8b). The vertical cross session of ocean temperature and current in the upper 150‐m depth passing this region (line CD in Figure 9a) is plotted in Figure 11d. The corresponding temperature difference (DON‐DOFF) shows that the dust‐induced warm temperature can be observed in the entire upper‐ocean layer (150‐m depth in Figure 11e), and is not limited near the surface, due to the dust‐induced enhanced SDRF. However, we note that the dust‐induced changes of surface and near‐surface properties (e.g., SST and 2‐m temperature and moisture) over this region (25–35°N) are sensitive to whether dust coverage is extended to this area. For example, the SST (Figure 9b) and 2‐m temperature and moisture (figure not shown) changes by dust over this region are negative in the doubled dust emission experiment, opposite to those in DON.

**Figure 12:**
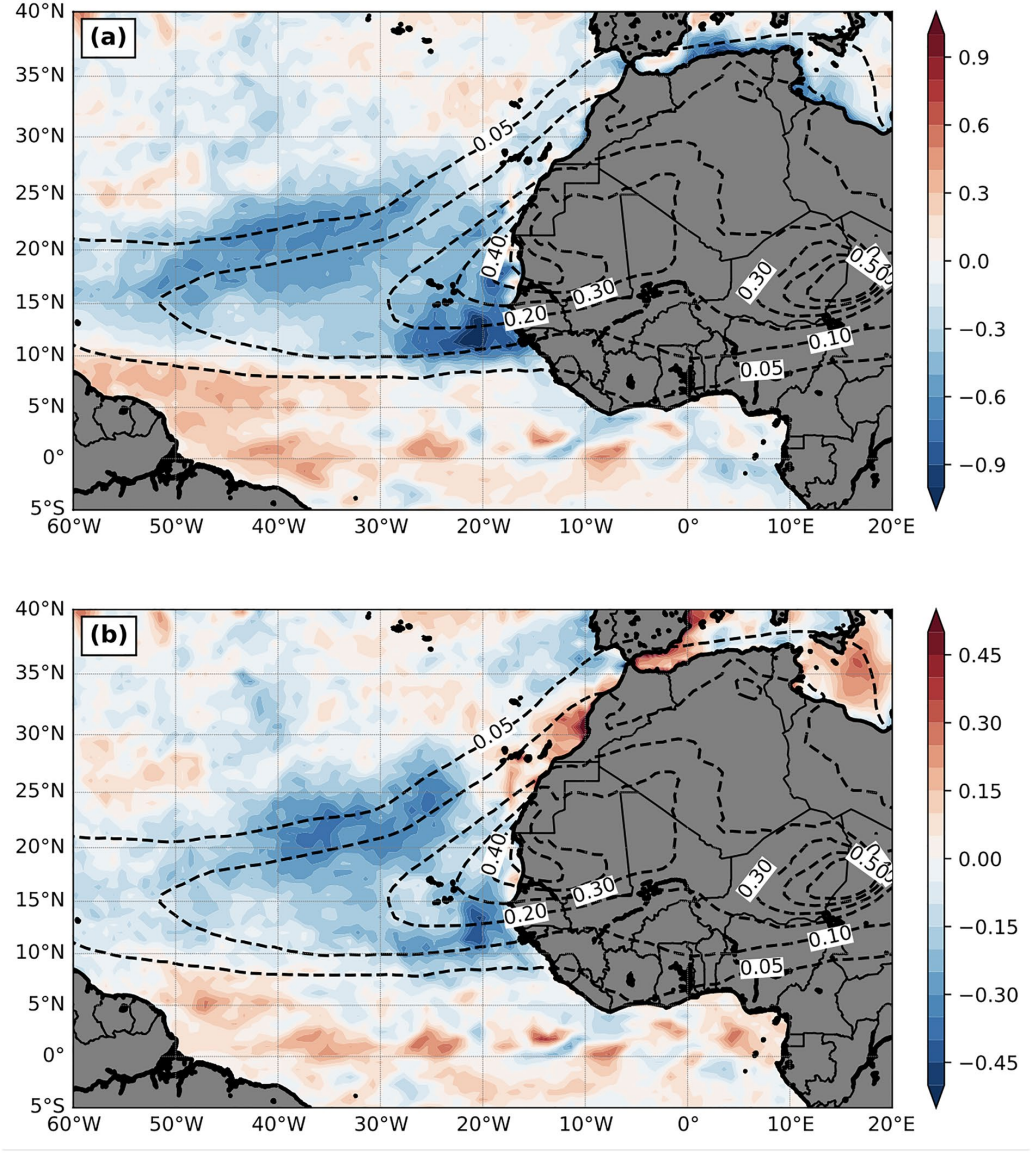
The differences between DON and DOFF (DON—DOFF) of their 3‐month mean (July‐September, 2015) (a) moisture dissimilarity between sea surface saturation mixing ratio (q_s_(SST)) and 2‐m water vapor mixing ratio (q(2‐m)) (q_s_(SST) − q(2m); unit: g kg^−1^) and (b) temperature dissimilarity between SST and 2‐m air temperature (T(2m) (SST − T(2m); unit: K). (b) is the same as the difference between Figures 9a and 7b (Figure 9a − Figure 7b). The dashed contours are the AOD field from DON.

For dust‐induced LHF and SHF changes, although they both are negative underneath the dust plume (except the SHF over the offshore region of northwestern North Africa), the causes of the changes to each flux are different. For LHF, the maximum negative change pattern between 10°N and 15°N is almost collocated with the weakening region of surface winds by dust (Figure 7a), a dominant factor for the LHF change. In addition to 10‐m winds, the dust‐induced change of moisture difference between the surface and 2 m (Figure 12a) also contributes to the LHF change. In particular, the large negative moisture difference induced by dust at the southern dust plume near the coast (Figure 12a) also plays an important role in the negative LHF change. Interestingly, the negative LHF change over this region coincides with the increase, instead of decrease, of 2‐m moisture by dust (Figure 7c). This is mainly due to the northward shift of ITCZ, which includes a convergence anomaly zone over this region (east of 30°W between 11°N and 16°N) as shown in the wind change in Figure 7a. For the region of 30–50°W and 15–20°N, the almost zero LHF change results from the compensation between the enhanced wind speed by dust (Figure 7a) over this region and the negative change of moisture difference between the surface and 2 m (Figure 12a).

Changes to SHF values occur mainly underneath the dust plume, with the pattern of anomalies resembling the plume shape. In addition to the cause of weaker 10‐m winds by dust over the southern half region of the dust plume as noted in the LHF changes, the reduction of SHF also occurs at the northern dust plume region which is dominated by the dust‐induced decrease of temperature between the surface and 2 m (Figure 12b). To the south of the dust plume (south of 10°N), both positive LHF and SHF released to the atmosphere (Figures 10a and 10b) are due to the dust‐induced SST warming, as well as stronger 10‐m winds east of 40°W. The ocean warming directly affects the surface moisture and temperature differences in the air‐sea bulk formula calculation (Figure 12), leading to the consequent changes to LHF and SHF values.

In summary, our results suggest that the atmospheric dust can mostly force SST changes through modifications of surface radiation and heat fluxes, near‐surface wind speed and direction, and/or the WSC. In some regions, the ocean and atmosphere can positively interact causing a more complicated SST response. For example, the eastern Atlantic ocean can positively feedback to the atmospheric circulation where a large change of SST cooling can be found in the atmospheric response; the most notable exception being a small nearshore region between 15°N and 20°N just underneath the maximum dust plume (Figure 9a). Also, the unexpected dust‐induced SST warming is induced by different mechanisms which combine to overcome the SST cooling by the reduction of SDRF. These results with high‐resolution coupling features are more complicated than those described using the coarse‐resolution global coupled model (e.g., Jordan et al., 2018; Strong et al., 2015).

## 5 Summary

Air‐sea interactions play a pivotal role in driving weather and climate, and these interactions are strongly modulated by radiative forcing, aerosol and cloud activities, large‐scale atmospheric and oceanic dynamics, and near‐surface meteorological and upper‐ocean variables. The Sahara Desert over North Africa emits the largest amount of dust aerosol in the world (Prospero et al., 2002) and its emission activity peaks during the summertime when the majority of dust plumes transport downstream to the Atlantic Ocean by AEJ and AEWs. Dust interacts with radiation and clouds, leading to the change of SDRF. In addition, these dust‐radiation‐cloud interactions can alter atmospheric thermal and dynamic properties, modifying near‐surface winds and upper‐ocean current to further affect the SST and air‐sea interactions.

This study investigates the influence of Saharan dust on summertime SST and air‐sea energy exchanges (i.e., LHF and SHF) over the Atlantic using a fully coupled AWOD regional model. The high‐resolution AWOD model integrates two existing models: the COAWST model (Warner et al., 2008, 2010) and the WRF‐Dust model (Chen et al., 2010, 2015). Two identical numerical experiments are conducted using the AWOD model, except with (DON) and without (DOFF) the activation of dust‐radiation‐cloud interactions. The model integrates for four months starting from June 1, 2015 and the last three months are used for analysis. We note that the computation is costly (more than 8 h for 1‐month simulation using 1,300 cores) so that the real‐time forecast is unrealistic. Longer simulation to investigate the dust impact on interannual or even decadal climate variabilities is possible, such as the work similar to Strong et al. (2015) and Jordan et al. (2018). However, this is beyond the scope of this study.

In general, the DON simulation shows much closer large‐scale patterns to the satellite AOD and reanalysis data than does the DOFF simulation. However, the DON simulation still underestimates the AOD within the entire domain. The DON simulation result shows slightly cold SST biases over the warm SST regions (western Atlantic and warm SST band along 10°N), warm SST biases at the cold SST regions (eastern Atlantic), a stronger subtropical high with a slightly southward shift in position, and weaker southerly and southeasterly equatorial flow. Considering that it is a 4‐month free simulation (June to September), the AWOD model actually performs reasonably well for the dust sensitivity study.

**Figure 13:**
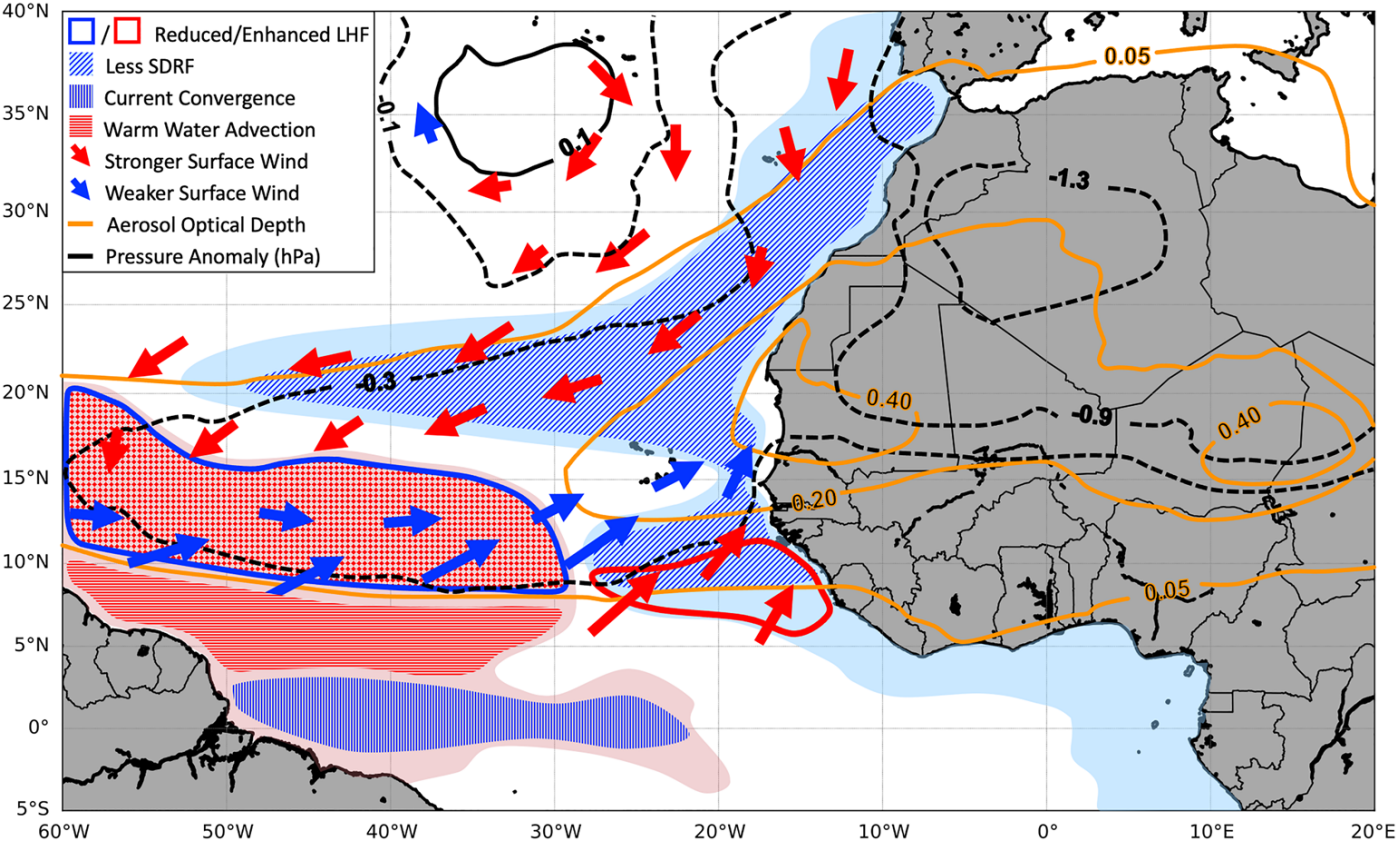
The schematic diagram of major dust‐induced mechanisms that are responsible for SST changes. The light blue (pink) shading is cold (warm) SST anomaly by dust. The thick solid (positive) and dashed (negative) black contours are dust‐induced SLP anomalies. The thick red (stronger) and blue (weaker) arrows are dust‐induced near‐surface wind anomalies. The orange contours indicate dust AOD. The diagonally hatched lines are dust‐induced changes of SDRF. The vertically hatched blue lines are dust‐induced convergence at the upper‐ocean layer. The horizontally hatched red lines indicate the dust‐induced warm water advection.

The comparison between DON and DOFF shows that in a 3‐month average, the dust radiative effects reduce the maximum net downward SW radiation flux by about 45 W m^−2^ and increase the maximum downward LW radiation flux by about 20 W m^−2^, with a maximum total reduction of about 20–30 W m^−2^. Dust increases SDRF north of the dust plume between 15°W and 35°W due to the reduction of LLCs by dust (blue cross‐hatched lines in Figure 6b). In addition to the influence of SDRF over the ocean, the dust‐radiation‐cloud interaction produces a surface low‐pressure anomaly underneath the dust plume (black dashed contours in the schematic diagram of Figure 13), which induces a cyclonic circulation difference surrounding the dust plume (large arrows in Figure 13); warms and moistens the air at 2 m along the dust plume (maximized at southern dust plume) and away from the coast of North Africa; and lowers 2‐m temperature and moisture fields offshore of the northwestern and southwestern coasts of North Africa.

In addition to the atmosphere, dust also significantly modifies the upper‐ocean layer. Dust produces a positive WSC anomaly under the dust plume region due to the cyclonic circulation anomaly near the surface with the maximum WSC anomaly aligning with the dust plume ridge. Dust produces a negative WSC anomaly at northern and southern edges of the dust plume due to the anticyclonic circulation anomaly. Dust also produces a positive WSC anomaly (∼30°N) south of the positive pressure anomaly of the intensified subtropical high. Dust decreases SSH and MLD under the main dust plume region with larger magnitudes over the western and southwestern side; dust increases SSH and MLD at the southern edge of the dust plume, which is generally consistent with the changes of surface WSC and upper‐ocean divergence by dust. To the south of the dust plume, dust induces a deeper MLD (i.e., positive anomaly), where convergence occurs at the upper‐ocean layer.

Patterns in SST changes from the presence of dust is very different from the dust plume shape. Moreover, atmospheric dust changes SST asymmetrically with respect to the dust plume. Dust cools SST near the coastal area of West Africa, except where the dust plume ridge is. The dust‐induced cold SST anomaly extends westward along the northern and southern edge of the dust plume and the northern branch extends farther west (55°W) compared to that of the southern branch (32°W). Dust unexpectedly warms SST over the west at the downstream of the dust plume and maximizes at the southern side of the dust plume. The influence of dust on SST also extends to areas beyond the dust plume. South of the dust plume, the warm SST anomaly at the downstream of the dust plume extends farther equatorward west of 30°W. Just north of the dust plume, a positive dust‐induced SST anomaly occurs between 20°W and 40°W due to the increase of SDRF. However, a cold SST change is obtained if this region is covered by dust after the dust emission is doubled.

The dust‐induced negative LHF and SHF change patterns are more in line with the dust plume pattern and thus are quite different from that of SST. The negative (downward) flux anomalies occur mainly underneath the dust plume region. However, their change patterns underneath the dust plume are different in detail. The change of LHF is more consistent with the wind weakening pattern by dust. The negative SHF change is more evenly distributed underneath the dust plume, except a positive SHF anomaly off the northwestern coast of West Africa, and the changes are controlled by dust‐induced changes of both near‐surface wind and temperature difference between SST and 2‐m atmosphere. The dust's influence on LHF and SHF is also beyond the dust plume coverage area. More negative SHF and LHF anomalies occur to the north of the dust plume and more positive anomalies to the south of the dust plume.

The above dust‐induced mechanisms that are responsible for SST changes are summarized in Figure 13. The SST cooling (light blue shading) by dust near the coast regions of West Africa is in part due to the reduction of SDRF (blue diagonally hatched). In addition, the dust‐induced intensification of upwelling due to the stronger upwelling‐favorable winds off northwestern West African coast (red arrows) and the dust‐induced enhancement of surface upward LHF (red thick contour) off southwestern West African coast are partially responsible for SST cold anomaly. The SST warming by dust (light pink shading) over the southwestern dust plume is mainly due to the reduction of surface trade winds (blue arrows) and thus surface LHF (blue thick contour), which stabilizes the upper‐ocean layer and reduces MLD (i.e., easier to heat up the SST). The SST warming near the equator is due to the increase of convergence at the upper‐ocean layer by dust (blue vertically hatched). Lastly, the SST warm change (light pink shading) to the south of the dust plume and the western tropical Atlantic is due to southwesterly 10‐m wind change by dust, which induces near‐surface southeastward current change and advects warm water to this region (red horizontally hatched). Finally, we would like to note that our current results are based mainly on one season. Studies from multiple warm seasons should be conducted in the future.

## Data Availability

The COAWST model is available https://www.usgs.gov/software/coupled-ocean-atmosphere-wave-sediment-transport-coawst-modeling-system. Several data sources were used in this study: CFSv2 global analysis data from https://rda.ucar.edu/datasets/ds094.0/; HYCOM reanalysis from https://www.hycom.org/dataserver/gofs-3pt0/analysis; MODIS AOD data from the NASA Distributed Active Archive Center (DAAC) web site. The modeled outputs resulting from these simulations are available upon request.
